# Supplementary material for: AAV5-miHTT Lowers Huntingtin mRNA and Protein without Off-Target Effects in Patient-Derived Neuronal Cultures and Astrocytes
Source: Mol Ther Methods Clin Dev. 2019 Oct 4;15:275–84. doi: 10.1016/j.omtm.2019.09.010 (PMC6849441; doi:10.1016/j.omtm.2019.09.010)
Supplement: Document S1. Figures S1–S6, Tables S1–S4, and Supplemental Materials and Methods [file mmc1.pdf]

OMTM, Volume *15*

Supplemental Information

AAV5-miHTT Lowers Huntingtin mRNA and Protein without Off-Target Effects in Patient-Derived Neuronal Cultures and Astrocytes

Sonay Keskin, Cynthia C. Brouwers, Marina Sogorb-Gonzalez, Raygene Martier, Josse A. Depla, Astrid Vallès, Sander J. van Deventer, Pavlina Konstantinova, and Melvin M. Evers

Supplementary Figure 1. Schematic of neuronal differentiation of induced-pluripotent stem cells (iPSC). To acquire HD patient-derived neuronal cultures, fibroblasts from HD patients with 71 CAG repeats (HD71, shown) or 180 CAG repeats (HD180, not shown) were reprogrammed to iPSC and subsequently differentiated into frontal brain-like neuronal cells. iPSC colonies were isolated and induced to neural progenitor cells by rosette isolation and neural tube formation. Neural tubes were selected and differentiated to neuronal precursors. Neuronal precursors were matured for 2 weeks.

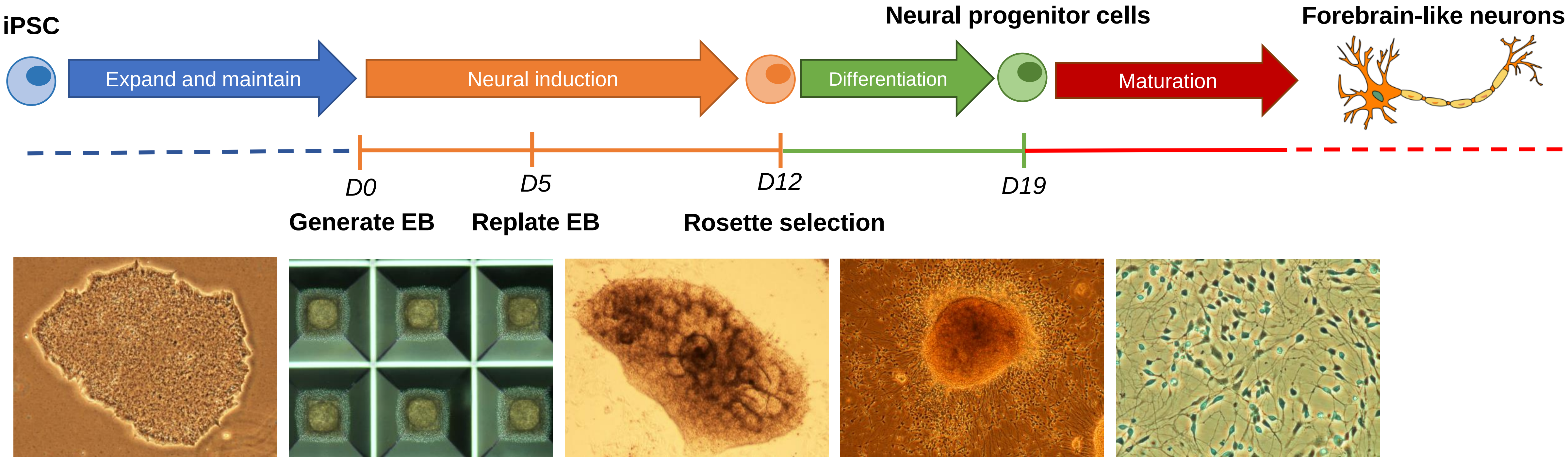

Supplementary Figure 2. Characterization of neuronal population. Characterization of HD iPSC-derived neuronal cultures by immunohistochemistry with antibodies detecting MAP2 and GFAP. A, E) DAPI staining for cell nuclei. B, F) MAP2 staining for neuronal cells. C, G) GFAP staining for astrocytes. D, H) Merged image of DAPI, MAP2 and GFAP staining showed the neuronal population is a mixture of neurons and astrocytes.

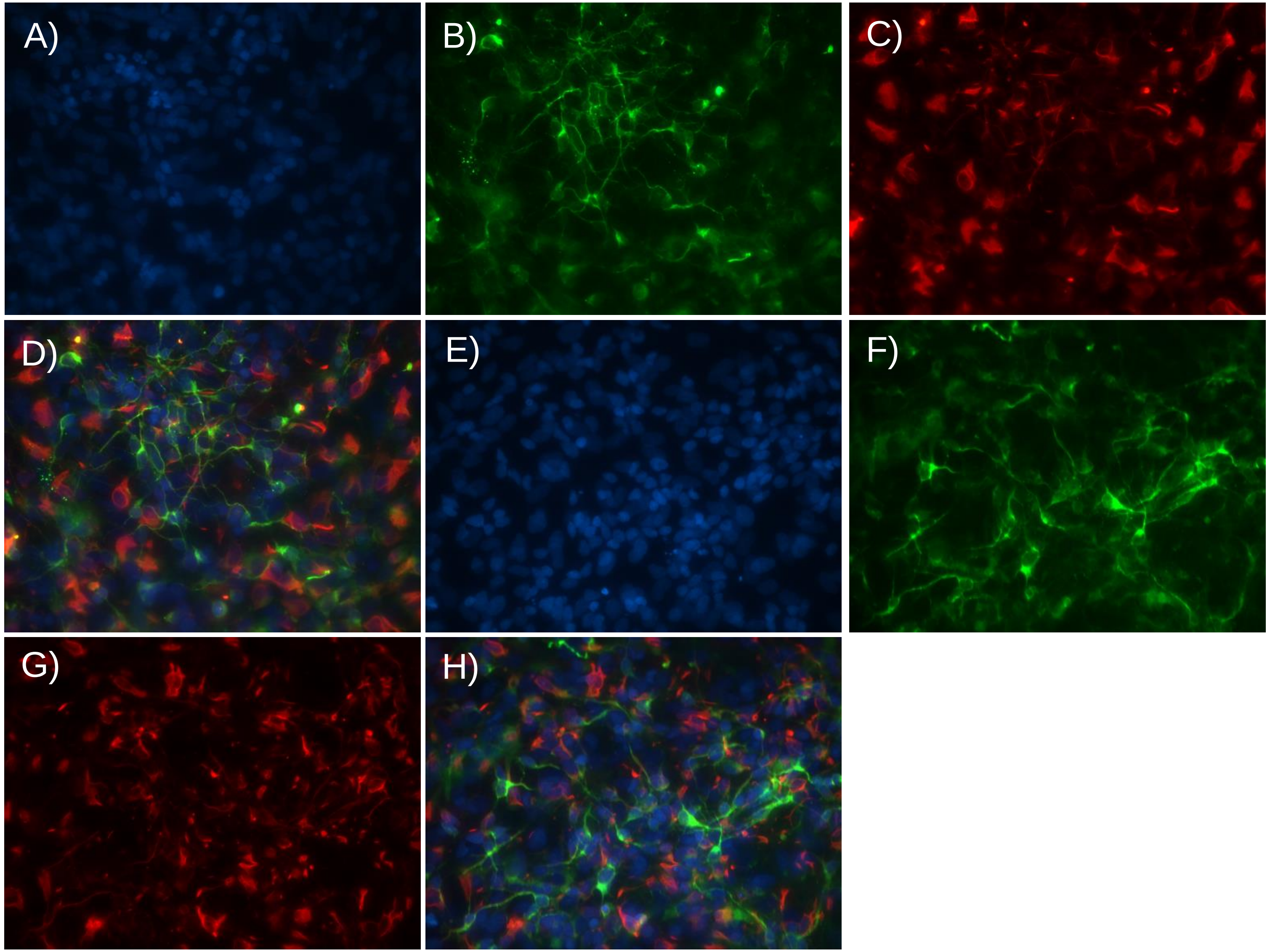

Supplementary Figure 3. Neuronal morphology of HD71 iPSC-derived neuronal cultures after 10 days of transduction. HD patient iPSC-derived neuronal culture treated with AAV5-miHTT MOI 10^5, MOI 10^7, AAV5-GFP MOI 10^7 and formulation buffer as control (see Supplementary Figure 3). Cells are kept in culture for 10 days after transduction and medium was refreshed twice a week. T=D0) pictures of neuronal culture before transduction. T=D4) pictures of neuronal culture taken 4 days after transduction. T=D7) pictures of neuronal culture taken 7 days after transduction. T=D10) pictures of neuronal culture taken 10 days after transduction.

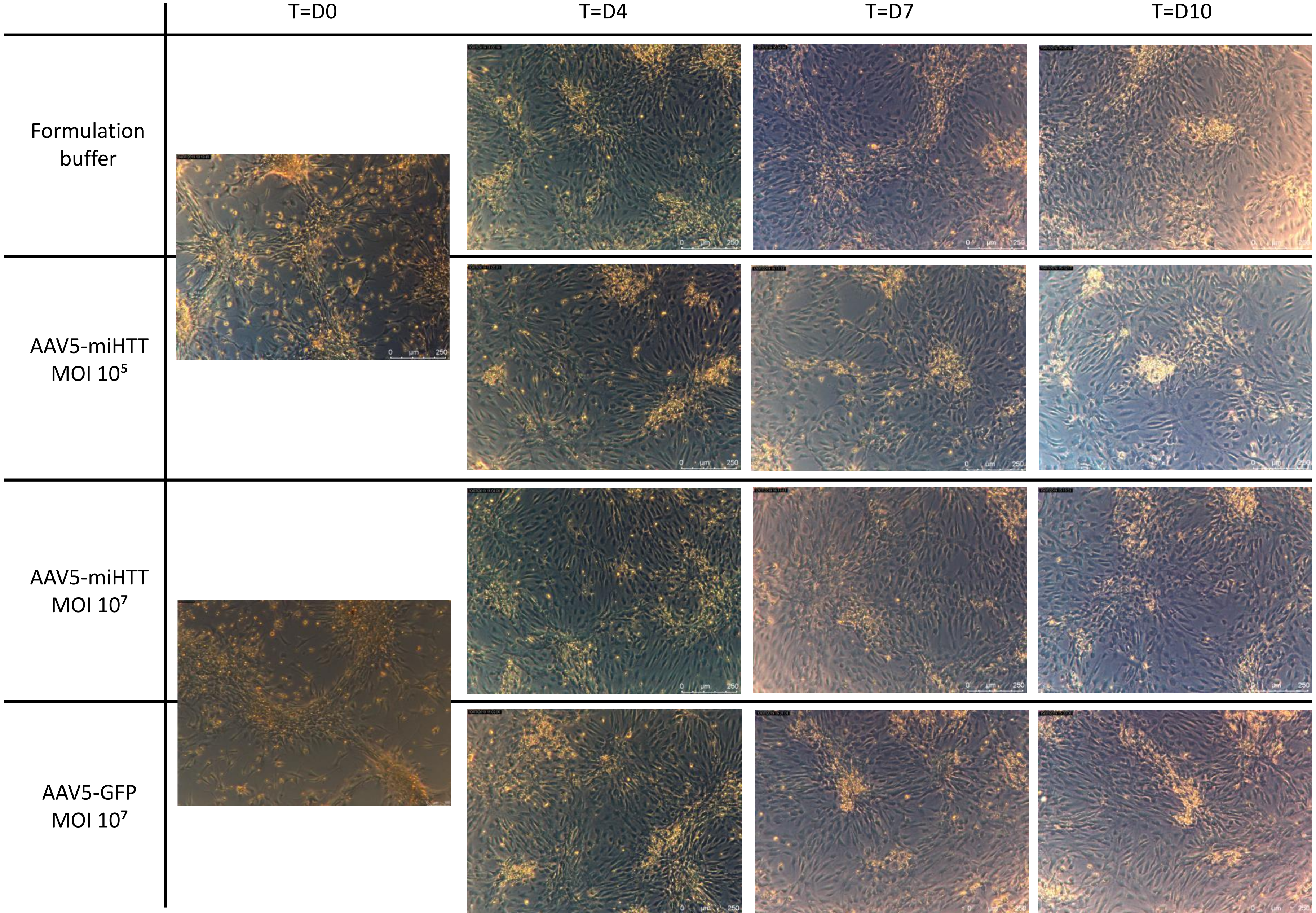

Supplementary Figure 4. Small RNA sequencing analysis of miHTT processing in HD patient iPSC-derived neuronal culture. Most abundant mature miHTT guide strands with sequence distribution in %. The *in-silico* predicted guide strand was 22 nucleotides in length.

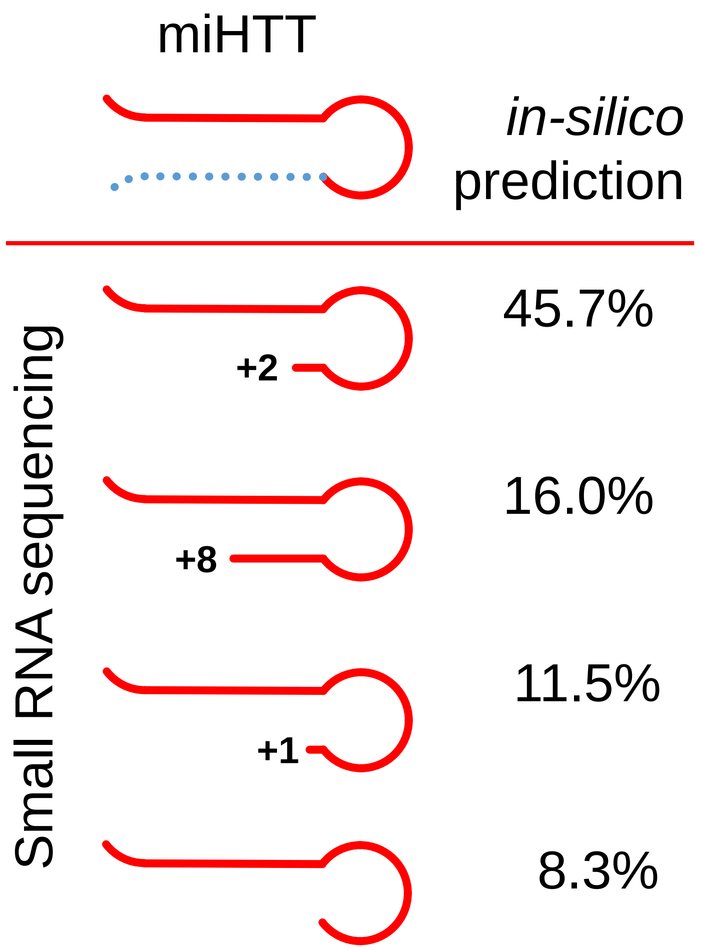

Supplementary Figure 5. Venn diagrams of RNA sequence comparisons of treatment groups. To investigate miHTT-related total transcriptome changes and gene pathway changes, Ingenuity Pathway Analysis (IPA) was performed to compare differentially expressed genes following AAV5-miHTT, AAV5-GFP and formulation buffer treatment.

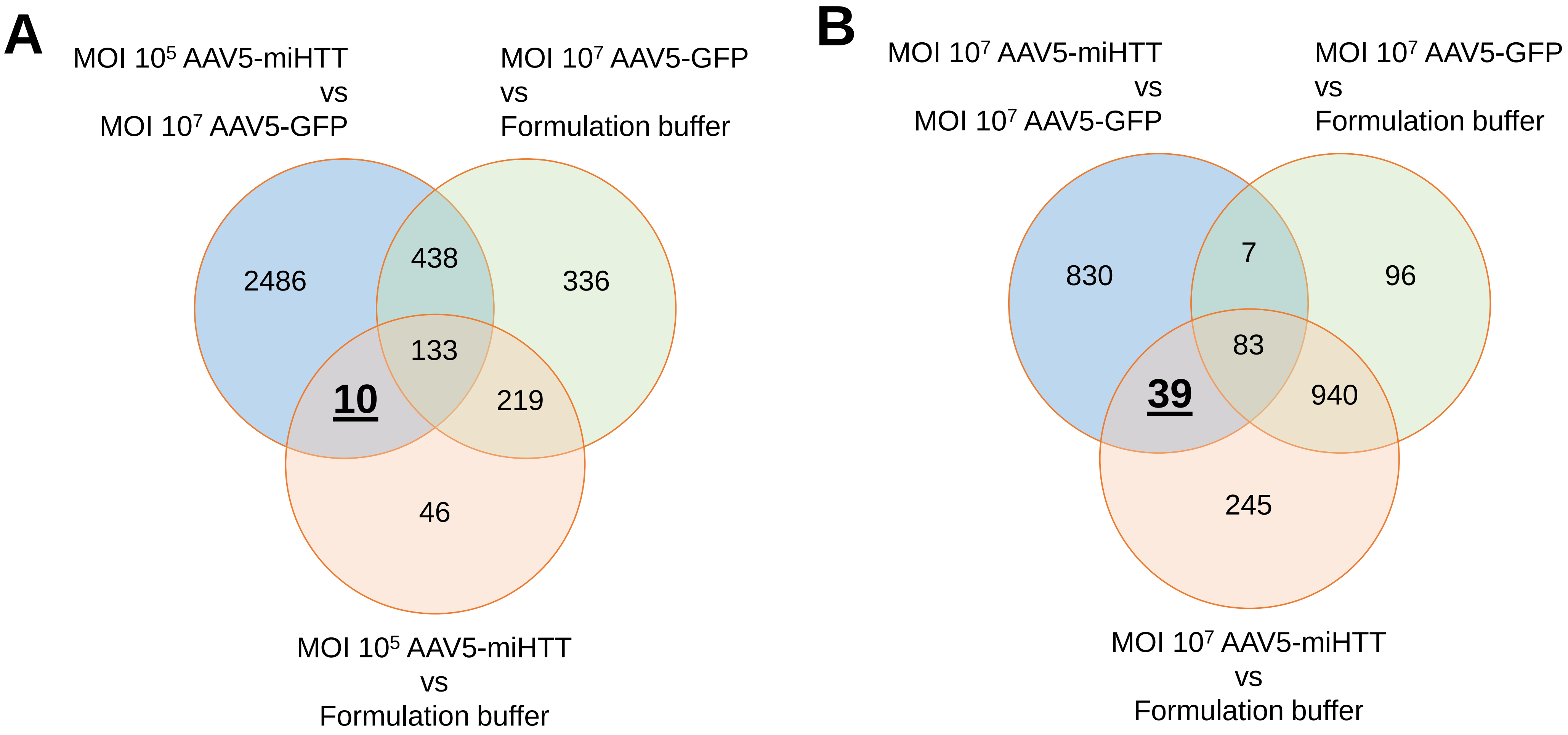

Supplementary Figure 6. Characterization of mature iPSC-derived astrocytes. Mature iPSC-derived astrocytes were fixed and stained for β tubulin III, tyrosine hydroxylase and GFAP. A, D, G) DAPI staining of astrocytes. B) Astrocytes were negative for β tubulin III. C) Merged image of DAPI and β tubulin III. Cells were negative for β tubulin III in all cells. E) Astrocytes were negative for tyrosine hydroxylase. F) Merged image of DAPI and tyrosine hydroxylase showed that zero cells were positive for tyrosine hydroxylase. H) Astrocytes were GFAP+. I) Merged image of DAPI and GFAP showed that all cells are positive for GFAP.

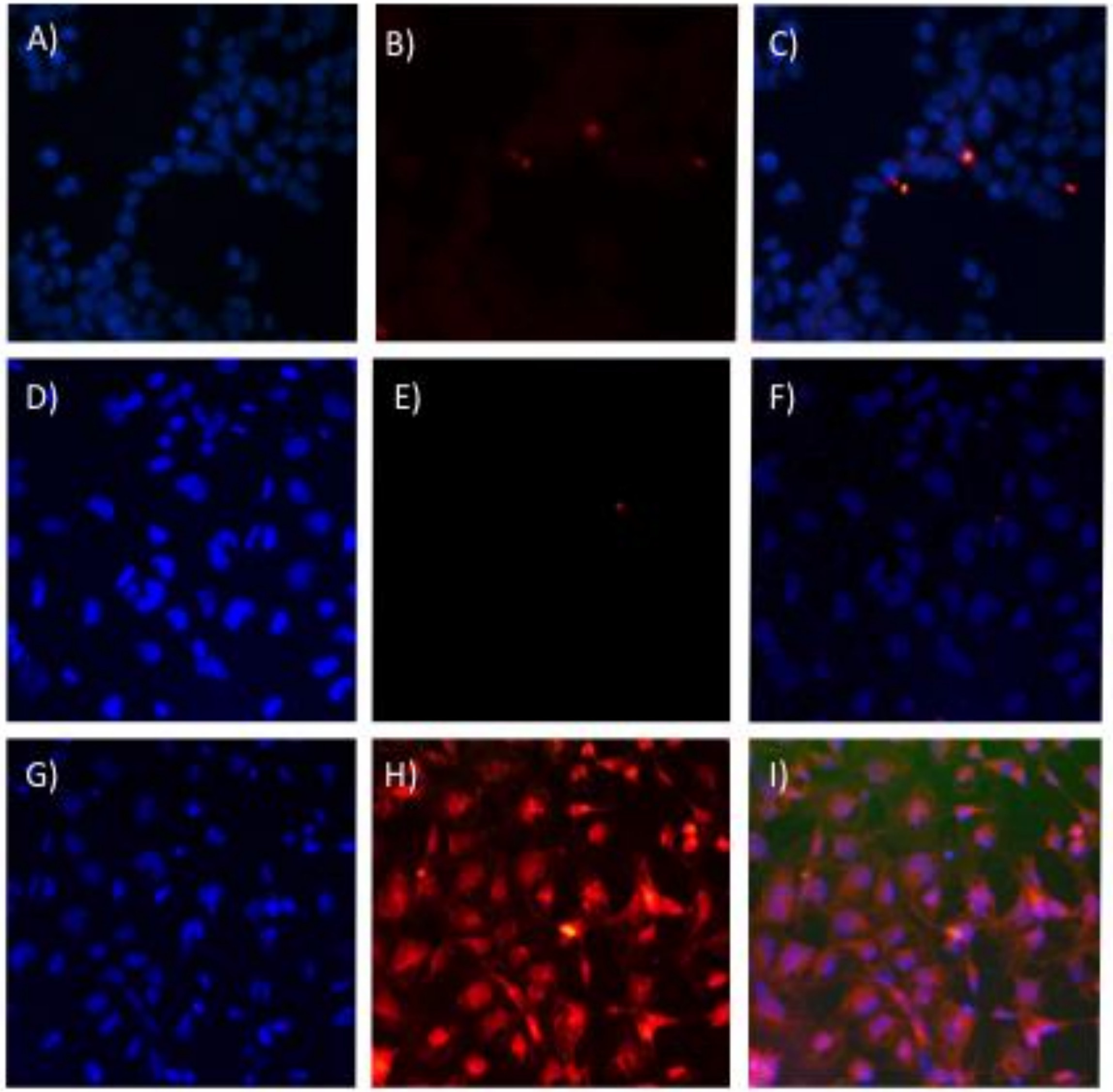

Supplementary Table 1. Target genes of the 10 differentially expressed miRNAs. For the 10 differentially expressed miRNAs the target genes are shown according to BLAST and siSPOTR.

miRNA	miRbase naming	Sequence miRbase	Mature reads	Target gene BLAST	Target gene siSPOTR
Let-7C	hsa-let-7c-5p MIMAT0000064	5'- UGAGGUAGUAGGUUGUAUGGUU -3'	6,84E+07	*HMGCR*	*IGF2BP1*
Mir-26a-1	hsa-miR-26a-5p MIMAT0000082	5'- UUCAAGUAAUCCAGGAUAGGCU -3'	2,85E+07	*CHST9*	*POLH*
Mir-26a-2	hsa-miR-26a-5p MIMAT0000082	5'- UUCAAGUAAUCCAGGAUAGGCU -3'	2,85E+07	*CHST9*	*POLH*
Mir-34a	hsa-miR-34a-5p MIMAT0000255	5'- UGGCAGUGUCUUAGCUGGUUGU -3'	1,56E+05	*SLC13A1*	*HCN3*
Mir-199a-1	hsa-miR-199a-5p MIMAT0000231	5'- CCCAGUGUUCAGACUACCUGUUC -3'	1,83E+06	*TRIP11*	*ZNF763*
Mir-199a-2	hsa-miR-199a-5p MIMAT0000231	5'- CCCAGUGUUCAGACUACCUGUUC -3'	1,83E+06	*TRIP11*	*ZNF763*
Mir-199b	hsa-miR-199b-5p MIMAT0000263	5'- CCCAGUGUUUAGACUAUCUGUUC -3'	7,08E+05	*TRIP11*	*ZNF763*
Mir-431	hsa-miR-431-5p MIMAT0001625	5'- UGUCUUGCAGGCCGUCAUGCA -3'	2,24E+02	*RTL1*	*PLEKHG4B*
Mir-450b	hsa-miR-450b-5p MIMAT0004909	5'- UUUUGCAAUAUGUUCCUGAAUA -3'	1,29E+04	*TM2D3*	*RBMS3*
Mir-503	hsa-miR-503-5p MIMAT0002874	5'- UAGCAGCGGGAACAGUUCUGCAG -3'	5,95E+04	*FOXK1*	*RASEF*

Supplementary Table 2. Transcript expressions in fold change compared to formulation buffer, after AAV5-miHTT and AAV5-GFP treatment, with corresponding P-values. For all target genes we have assessed the transcript expression in HD71 patient iPSC-derived neuronal cultures by RNAseq. Shown are the weighted proportions fold changes in transcript expression after transduction with AAV5-miHTT MOI 10^7 and AAV5-GFP MOI 10^7 compared to formulation buffer treatment, calculated by Baggerley's test. Also, the P-values are shown, calculated by Baggerley's test.

		AAV5-miHTT MOI 10^7 vs Formulation buffer		AAV5-GFP MOI 10^7 vs Formulation buffer	
	**Target gene**	**Fold change**	**P-value**	**Fold change**	**P-value**
**BLAST**	*HMGCR*	-1,2429	0,2188	-1,1564	0,4061
	CHST9	1,8327	0,5325	2,0164	0,4642
	SLC13A1	1	0,9310	1	0,9310
	TRIP11	1,0445	0,8973	1,0847	0,8094
	RTL1	**-2,8416**	**0,0001**	**-2,2398**	**0,0015**
	TM2D3	-1,0941	0,6772	-1,0492	0,8236
	FOXK1	-1,0226	0,9750	-1,0772	0,9185
**siSPOTR**	*IGF2BP1*	-1,0201	0,9347	-1,0333	0,8941
	POLH	2,4418	0,0272	2,2163	0,0565
	HCN3_1	-1,5359	0,7659	-1,2696	0,8630
	HCN3_2	-1,5004	0,7344	-1,6231	0,6934
	ZNF763	-1,0689	0,9862	1,7740	0,8636
	PLEKHG4B	-1,0017	0,9986	-1,0117	0,9909
	RBMS3	-1,4105	0,4023	-1,2376	0,5941
	RASEF	-1,7804	0,9187	-1,0322	0,9949

Supplementary Table 3. SYBR Green qPCR primer sequences

Gene	ENSEMBL transcript #	Fw Primer name	Sequence (5' - 3')	Length	Tm	GC%	Rv primer name	Sequence (5' - 3')	Length	Tm	GC%	Product length (bp)
LETM1	ENST00000302787.2	hLETM1_Fw1	ATGGATCGACACCAAGATCG	20	60,89	50,00	hLETM1_Rv1	ACCACCACGAACACAAGGA	19	59,99	52,63	141
RFFL	ENST00000315249.11	hRFFL_Fw2	CAACCTCCCCTCTTCATCTG	20	59,65	55,00	hRFFL_Rv2	AGACGGGTTCCTCTTGATCC	20	60,46	55,00	107
ALDH18A1	ENST00000371224.6	hALDH18A1_Fw1	AAGCATGCCAAGAGAATCGT	20	59,84	45,00	hALDH18A1_Rv1	CACCAGCATCATCTCTCTGC	20	59,53	55,00	144
PGPEP1	ENST00000269919.10	hPGPEP1_Fw1	GTGGGAGAAGCACAGTCCAC	20	60,72	60,00	hPGPEP1_Rv1	TGTAGCCCTTGTTGTGTCCA	20	60,15	50,00	146
ODF2	ENST00000351030.7	hODF2_Fw1	CCCCCTGGAAAATCATCTG	19	60,26	52,63	hODF2_Rv1	TAACCGCATCACTGAGACCA	20	60,26	50,00	111
ADGRA2	ENST00000412232.2	hADGRA2_Fw1	CCCGGCCTTCTGCCTAAC	18	62,99	66,67	hADGRA2_Rv1	AGTGACAGTCCCAGGAAGGA	20	59,68	55,00	92
ZNF596	ENST00000308811.8	hZNF596_Fw1	TTCCCAATTGGAGCAAGTAGA	21	59,69	42,86	hZNF596_Rv1	GTGGACGTGCCCTTCTGATA	20	61,07	55,00	126
KIFC1_1	ENST00000428849.6	hKIFC1_1_Fw1	TGCAACGACCAAAATTACCA	20	59,97	40,00	hKIFC1_1_Rv1	CTGTGGCAATAGCTGTGGAA	20	59,86	50,00	134
GFRA1	ENST00000369236.5	hGFRA1_Fw1	TGGAGGATTCCCCATATGAA	20	60,09	45,00	hGFRA1_Rv1	TTGTTCCCTTTGGGAATGTG	20	60,72	45,00	97
RUBCN	ENST00000273582.9	hRUBCN_Fw1	CTGGAAGCAGTGGAACAGAA	20	59,01	50,00	hRUBCN_Rv1	CTGTCAGGCTCTGGCTCTTT	30	59,75	55,00	112
APOL6	ENST00000409652.4	hAPOL6_Fw1	ACACAGATTTGCTGCCACAG	20	59,90	50,00	hAPOL6_Rv1	GCTCCACGTCTTCACACAGA	20	60,03	55,00	114
SH3TC2	ENST00000515425.5	hSH3TC2_Fw1	TCCTTCCAAGGATCCAACTG	20	60,04	50,00	hSH3TC2_Rv1	GCGGCTCTTTACACAGAAGG	20	60,02	55,00	121
NRP2	ENST00000360409.7	hNRP2_Fw1	ACTACCCCTCCCACCAGAAC	20	60,23	60,00	hNRP2_Rv1	CCATCCCGAATCTCGATAAA	20	59,86	45,00	136
KIAA2022	ENST00000055682.10	hKIAA2022_Fw1	CAGCCAACGGAGAAAACACT	20	60,29	50,00	hKIAA2022_Rv1	CGGTGTAGGCTGGATAGGTG	20	60,53	60,00	119
CACNA1C	ENST00000399655.5	hCACNA1C_Fw1	CTGCATCAGCATTGTCGAAT	20	59,83	45,00	hCACNA1C_Rv1	GATCGCTAAGGCCACACAAT	20	60,10	50,00	82
CSRNP3	ENST00000342316.8	hCSRNP3_Fw1	AGTGGGGACAGTGTCAATCC	20	59,82	60,25	hCSRNP3_Rv1	CCTTGCCTCCTGGTGAAGTA	20	60,25	55,00	134
NEGR1	ENST00000357731.9	hNEGR1_Fw1	GATGGTCAGAAAAGGGGACA	20	59,90	50,00	hNEGR1_Rv1	CTTATCACCTCCCGCAAAAA	20	60,07	45,00	109
SYNCRIP	ENST00000369622.7	hSYNCRIP_Fw1	AGGACCAGATGAGGCAAAAA	20	59,67	45,00	hSYNCRIP_Rv1	AACAGAAGGCTGCTGACCTG	20	60,59	55,00	130
ST8SIA1	ENST00000396037.8	hST8SIA1_Fw1	TACATCTTCCCCGTCTACCG	20	59,95	55,00	hST8SIA1_Rv1	GTCGCAGCAGTCTTCCATTT	20	60,41	50,00	132

Gene	ENSEMBL transcript #	Fw Primer name	Sequence (5' - 3')	Length	Tm	GC%	Rv primer name	Sequence (5' - 3')	Length	Tm	GC%	Product length (bp)
CALCRL	ENST00000392370.7	hCALCRL_Fw1	TGGACACGGATTGTCTATTGC	21	60,91	47,62	hCALCRL_Rv1	AATCCTTTGGCAACTTAGGC	20	58,33	45	82
LIX1	ENST00000274382.8	hLIX1_Fw1	GCCTCCCTTTGTGAGTTACG	20	59,73	55	hLIX1_Rv1	GATCAGGGCCACTTTAGCTG	20	59,84	55	112
DDIT4L	ENST00000273990.6	hDDIT4L_Fw1	TGCTGGACTGTGGCTATCAC	20	59,86	55	hDDIT4L_Rv1	GAGGTTGGGTTCAGGAACAA	20	59,94	50	80
RERG	ENST00000256953.6	hRERG_Fw1	GATTTCTGACCAAACGGTTCA	21	59,96	42,86	hRERG_Rv1	ATCATCGATGGTTGCTTGGT	20	60,35	45	80
ADGRF5	ENST00000265417.7	hADGRF5_Fw1	TGAACAGCCTCAGTTTTCCA	20	59,41	45	hADGRF5_Rv1	GCAGGAGCACCAGATTTCAT	20	60,23	50	116
COMTD1	ENST00000372538.7	hCOMTD1_Fw1	AACTGCTCCGCCTACTACGA	20	60,04	55	hCOMTD1_Rv1	GTCCCCTTTCGGAGGTTG	18	60,44	61,11	111
RGPD4	ENST00000408999.3	hRGPD4_Fw1	GGTGGAAAGAGCAGCAAAAC	20	59,86	50	hRGPD4_Rv1	TCCATCCATCTTCACCTTCA	20	59,01	45	92
CLDN5	ENST00000403084.1	hCLDN5_Fw1	CTGGACCACAACATCGTGAC	20	60	55	hCLDN5_Rv1	CACCGAGTCGTACACTTTGC	20	59,36	55	105
DACH1	ENST00000611519.4	hDACH1_Fw1	AACCGCTGCAAACTCATCTC	20	60,41	50	hDACH1_Rv1	CTTAGGAGGCCTTCCAGGTC	20	60,2	60	90
ZFPM1	ENST00000319555.7	hZFPM1_Fw1	CTGGAAGGACAGGAACCAGA	20	60,23	55	hZFPM1_Rv1	CTCTGCCCATCCTGCAC	17	58,77	64,71	101
BACT	ENST00000331789,9	hACTBEx2FW1	GTCTTCCCCTCCATCGTG	18	58.2	61,1	hACTBEx3REV1	TCTTGCTCTGGGCCTCGT	18	58,2	61,1	97
HMBS	ENST00000278715.7	hHMBS_Fw2	CACCCACACACAGCCTACTTT	21	60,08	52,38	hHMBS_Rv2	GTACCCACGCGAATCACTCT	20	60,14	55	107

Supplementary Table 4. Top 15 hits *in-silico* off-target prediction miHTT-451 guide strand by siSPOTR

**Number**	**Gene ID**	**Gene**	**TPOTS**	**8mer**	**7mer-M8**	**7mer-1A**
**1**	80830	*APOL6*	0.596	2	3	1
**2**	79628	*SH3TC2*	0.508	1	4	1
**3**	2849	*GPR26*	0.434	1	2	2
**4**	51208	*CLDN18*	0.391	2	1	0
**5**	8828	*NRP2*	0.386	2	0	1
**6**	5241	*PGR*	0.385	2	0	1
**7**	340533	*KIAA2022*	0.367	1	2	1
**8**	775	*CACNA1C*	0.367	1	2	1
**9**	3977	*LIFR*	0.366	1	2	1
**10**	80034	*CSRNP3*	0.362	1	1	2
**11**	79068	*FTO*	0.355	1	0	3
**12**	257194	*NEGR1*	0.322	2	0	0
**13**	10492	*SYNCRIP*	0.321	2	0	0
**14**	6489	*ST8SIA1*	0.321	2	0	0
**15**	23007	*PLCH1*	0.321	2	0	0

Supplementary methods

Probability of Off-Target Score (POTS)

Provides summary information on the total number of predicted off-targets and the distribution of the various "site-types" found in those predicted targets. The POTS value is calculated by analyzing the potential off-target genes for seed matches conforming to known endogenous microRNA targeting rules. "8mer" sites tend to be the most potent followed by (in decreasing order) 7mer-M8, 7mer-1A and 6mer (https://sispotr.icts.uiowa.edu/sispotr/tools.html).

Potential off-target list

A list of probable off-target genes rank-ordered by individual transcript Probability of Off-Target Score (tPOTS). tPOTS was calculated based on the number and type of seed matches found in that transcript. "8mer" sites tend to be the most potent followed by (in decreasing order) 7mer-M8, 7mer-1A and 6mer (https://sispotr.icts.uiowa.edu/sispotr/tools.html).
