# Supplementary material for: AAV5-miHTT Lowers Huntingtin mRNA and Protein without Off-Target Effects in Patient-Derived Neuronal Cultures and Astrocytes
Source: Mol Ther Methods Clin Dev. 2019 Oct 4;15:275–84. doi: 10.1016/j.omtm.2019.09.010 (PMC6849441; doi:10.1016/j.omtm.2019.09.010)
Supplement: Document S2. Article plus Supplemental Information [file mmc2.pdf]

Molecular Therapy
Methods & Clinical Development
Original Article

AAV5-miHTT Lowers Huntingtin mRNA and Protein without Off-Target Effects in Patient-Derived Neuronal Cultures and Astrocytes

Sonay Keskin,[1] Cynthia C. Brouwers,[1] Marina Sogorb-Gonzalez,[1,2] Raygene Martier,[1,2] Josse A. Depla,[1,3] Astrid Vallès,[1] Sander J. van Deventer,[1,2] Pavlina Konstantinova,[1] and Melvin M. Evers[1]

[1]Department of Research & Development, uniQure biopharma, B.V., Amsterdam, the Netherlands; [2]Department of Gastroenterology and Hepatology, Leiden University Medical Center, Leiden, the Netherlands; [3]Department of Medical Microbiology - Clinical Virology, Amsterdam Medical Center, Amsterdam, the Netherlands

Huntington disease (HD) is a fatal neurodegenerative genetic disorder, thought to reflect a toxic gain of function in huntingtin (Htt) protein. Adeno-associated viral vector serotype 5 (AAV5)- microRNA targeting huntingtin (miHTT) is a HD gene-therapy candidate that efficiently lowers *HTT* using RNAi. This study analyzed the efficacy and potential for off-target effects with AAV5-miHTT in neuronal and astrocyte cell cultures differentiated from induced pluripotent stem cells (iPSCs) from two individuals with HD (HD71 and HD180). One-time AAV5-miHTT treatment significantly reduced human *HTT* mRNA by 57% and Htt protein by 68% in neurons. Small RNA sequencing showed that mature miHTT was processed correctly without off-target passenger strand. No cellular microRNAs were dysregulated, indicating that endogenous RNAi machinery was unaffected by miHTT overexpression. qPCR validation of *in silico*-predicted off-target transcripts, next-generation sequencing, and pathway analysis confirmed absence of dysregulated genes due to sequence homology or seed-sequence activity of miHTT. Minor effects on gene expression were observed in both AAV5-miHTT and AAV5-GFP-treated samples, suggesting that they were due to viral transduction rather than miHTT. This study confirms the efficacy of AAV5-miHTT in HD patient iPSC-derived neuronal cultures and lack of off-target effects in gene expression and regulation in neuronal cells and astrocytes.

INTRODUCTION

Huntington disease (HD) is a fatal neurodegenerative genetic disorder that affects motor function and leads to behavioral symptoms and cognitive decline in adulthood, resulting in total physical and mental deterioration over a 12- to 15-year period.[1,2] HD is caused by the expansion of CAG trinucleotides in exon 1 of a multifunctional gene coding for the huntingtin (Htt) protein.[2] The exact role of the mutant protein in the disease pathophysiology is not fully elucidated, but it is thought that a toxic gain of function is responsible for the initial development and progressive neurodegeneration in HD.[3] Based on the current preclinical data and hypotheses, reduction of mutant (toxic) *HTT* is anticipated to ameliorate disease progression, which has motivated various approaches to *HTT* lowering.

To lower HTT, we developed an adeno-associated viral vector serotype 5 (AAV5) that delivers a microRNA (miRNA) that targets human *HTT* mRNA (AAV5-miHTT). AAV5-miHTT is a one-time gene therapy candidate for HD based on the lowering of mutant *HTT* expression using RNAi.[4–8] The expression cassette for AAV5-miHTT consists of the cytomegalovirus immediate-early enhancer fused to chicken β-actin (CAG) promoter, the pri-miHTT expression cassette engineered in the pri-miR-451 backbone, and the human growth hormone poly(A) signal.[4] The AAV5-miHTT cassette is flanked by two intact noncoding inverted terminal repeats (ITRs) that originate from the wild-type AAV2 viral genome. In several rodent, minipig, and nonhuman primate studies, AAV5-miHTT has demonstrated preferential vector distribution and miRNA expression in the striatum, the primary site of neurodegeneration in HD, and in the sensorimotor cortex, also affected during disease progression.[6,8] This expression of therapeutic miHTT in key brain areas relating to HD results in lowering of *HTT* mRNA and mutant Htt protein, reduction in mutant Htt aggregates, and improvements in neuronal dysfunction, behavior, and survival in different small and large animal disease models.[5–8]

AAV5-miHTT was specifically designed to optimize *HTT* mRNA lowering while avoiding off-target effects. To eliminate a potential source of off-target effects, AAV5-miHTT was designed using the pri-miR-451 backbone so that when the miRNA is processed into the active guide strand there is no passenger-strand production.[4] The *HTT* mRNA lowering effect of miHTT is based on 21–23 nucleotide homology, so theoretically there is a potential that it might also bind and lower the expression of other genes. To investigate this, the current study examined putative off-target activity of AAV5-miHTT seed sequence, AAV5-miHTT and *HTT*-lowering specific effects, miHTT-related total transcriptome and gene pathway changes, and cell-type-specific effects. As off-target activity may be both species

Received 3 March 2019; accepted 30 September 2019;
https://doi.org/10.1016/j.omtm.2019.09.010.

Correspondence: Melvin M. Evers, Department of Research & Development, uniQure biopharma, B.V., Paasheuvelweg 25A, Amsterdam, the Netherlands.
E-mail: m.evers@uniqure.com

This is an open access article under the CC BY license (http://creativecommons.org/licenses/by/4.0/).

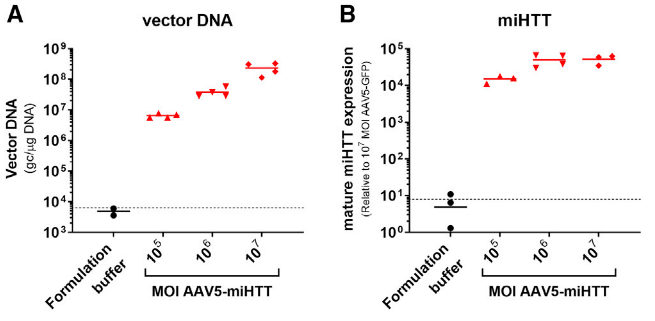

Figure 1. Dose-Dependent AAV5-miHTT Transduction and miHTT Expression in HD71 Patient iPSC-Derived Neuronal Culture

HD patient iPSC-derived neuronal culture was transduced with AAV5-miHTT at a MOI of 10^5, 10^6, or 10^7. (A) Ten days post-transduction, DNA was isolated and AAV5-miHTT vector DNA gc per µg gDNA was determined by SYBR Green qPCR. The dotted line reflects the lower limit of quantification for vector DNA. (B) Simultaneously, RNA was isolated and mature miHTT expression was determined relative to AAV5-GFP at a MOI of 10^7, using U6 as a reference gene. The dotted line reflects the miHTT assay background.

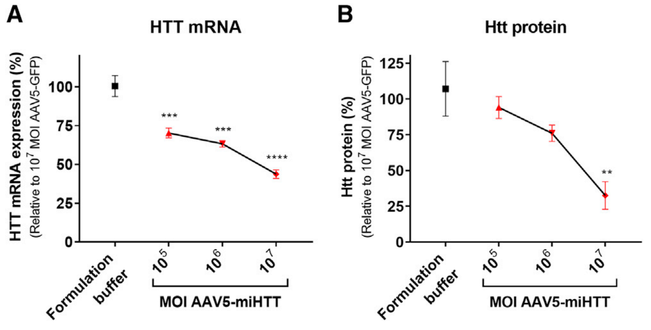

Figure 2. *HTT* mRNA and Mutant Htt Protein Lowering in HD71 Patient iPSC-Derived Neuronal Culture

(A) *HTT* mRNA levels relative to the mean expression in control-treated cells (AAV5-GFP at a MOI of 10^7) were determined by gene-specific TaqMan qPCR. (B) Ultra-sensitive single molecule counting assay with 2B7 and MAB2166 antibodies to quantify human Htt protein (both wild-type and mutant), relative to AAV5-GFP at a MOI of 10^7. Data were evaluated using a one-way ANOVA and corrected using a Bonferonni test. $^{**}p < 0.01$, $^{***}p < 0.001$, $^{****}p < 0.0001$ versus controls.

and cell type specific, these analyses were done in a cell system that as closely as possible resembles the human patient situation, that is, neuronal and astrocytic cell cultures derived from induced pluripotent stem cells (iPSCs) from two individuals with HD (Figure S1).

RESULTS

Dose-Dependent AAV5-miHTT Transduction and miHTT Expression in HD Patient iPSC-Derived Neuronal Cultures Leads to Human *HTT* mRNA and Htt Protein Lowering

The aim of this study was to examine the efficacy of AAV5-miHTT in human neural cells from two individuals with HD and to rule out potential off-target effects. To examine efficacy, iPSCs were differentiated into a mixed population of neuronal and astrocytic cells (Figure S1) by dual inhibition of SMAD signaling.[9] The neuronal population is a mixture of predominantly frontal brain-like neurons and astrocytes as determined by microtubule-associated protein 2 (MAP2) staining for neuronal cells and glial fibrillary acidic protein (GFAP) staining for astrocytes (Figure S2). Neuronal cultures were transduced with a MOI of 10^5, 10^6, or 10^7 for AAV5-miHTT and harvested 10 days post-transduction. In this disease-relevant system, AAV5-miHTT transduction resulted in a dose-dependent expression of vector DNA (Figure 1A) and subsequent dose-dependent expression of mature miHTT in HD patient iPSC-derived neuronal cultures (Figure 1B).

Consistent with vector DNA and mature miHTT expression, a dose-dependent reduction of HTT mRNA of up to 56.8% (Figure 2A) and a reduction in total Htt protein of up to 68% (Figure 2B) was observed compared with control-treated cells. These data indicate that human iPSC-derived neuronal cultures from HD patients can be effectively transduced by AAV5-miHTT, which results in significant lowering of human *HTT* mRNA and human Htt protein.

Also, we have examined the effects of AAV5-miHTT and AAV5-GFP treatment on the neuronal morphology of HD patient iPSC-derived neuronal cultures. No changes in neuronal morphology were seen between any of the conditions, showing that both AAV5-GFP and, specifically, AAV5-miHTT are well tolerated by HD iPSC-derived neuronal cultures (Figure S3).

Processing of miHTT in HD Patient iPSC-Derived Neuronal Cultures Results in Guide Strand Expression without Passenger Strand Activity and Saturation of the Endogenous RNAi Machinery

Having demonstrated the efficacy of AAV5-miHTT, we next investigated the processing of the therapeutic miHTT in human cells. The scaffold miR-451 that we used employs a dicer-independent pathway.[10,11] First, drosha cleaves the miR-451 precursor and is loaded into Ago2. Ago2 cleaves the opposite nucleotide 10–11 of the guide strand and produces the typical 30-nt fragment. This product is further trimmed by the poly(A)-specific ribonuclease (PARN) to the mature 22- to 26-nt miR-451.[12] The trimming is not an essential process, and mature miR-451, which is longer than the mature length, still remains functional.[13] Drosha processing at the 5′ end always results in the cleavage of a single-seed sequence. Thus, all processed guide strands have identical 3′ seed regions with identical on-target functionality and off-target activity. The main advantage of using the miR-451 precursor is the complete lack of a passenger strand processing and thus reduces off-target activity as compared to other scaffolds.[4] Small RNA sequencing showed that the mature miHTT was processed correctly without any passenger strand activity (Figure S4). The most abundant percentage form of the mature miHTT molecule was 24 nt long (45.7%). No miHTT-derived sequences aligned to the formulation buffer-treated control samples, indicating the specificity of the small RNA sequencing analysis. These data indicate that AAV5-miHTT has no risk of passenger-derived off-target effects. The lack of passenger strand formation and processing

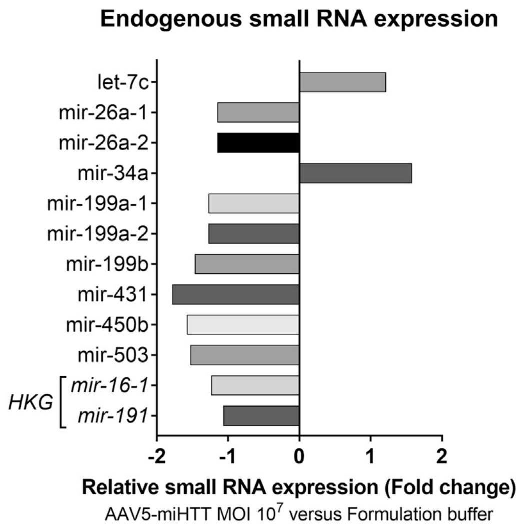

Figure 3. Small RNA Sequencing Analysis of AAV5-miHTT-Treated HD71 iPSC-Derived Neuronal Culture

HD71 patient iPSC-derived neuronal culture was transduced with AAV5-miHTT at a MOI of 10^7 (n = 4) and formulation buffer (n = 4) as control. Ten days after transduction, RNA was isolated and small RNA sequencing was performed. The reads were aligned to the human reference sequence. The small RNA reads of the AAV5-miHTT-treated samples were compared to the reads of the control samples (treated with formulation buffer-treated) to obtain the relative expression of endogenous small RNAs of treated versus control samples (cut-off false discovery rate p value <0.05). Relative expression was calculated and expressed as fold change compared with controls.

of miHTT to a 24-nt-long mature sequence supports its superior potential for high efficacy without off-target activity.

Two important aspects besides the processing and passenger strand formation of our therapeutic miRNA are (1) the potential changes in endogenous *miRNA* expression and (2) potential saturation of endogenous RNAi machinery by AAV5-miHTT (i.e., level of expression of miHTT with respect to endogenous miRNAs). To address these questions, small RNA sequencing was performed. The first analysis compared the small RNA reads of neuronal cultures from one of the HD patients (HD71) treated with either AAV5-miHTT at a MOI of 10^7 (n = 4) or formulation buffer (n = 4). After Bonferroni's correction, only 10 endogenous miRNA transcripts were found to be significantly differentially expressed after AAV5-miHTT transduction of HD iPSC-derived neuronal cultures. The changes in expression compared with formulation buffer-treated cells were small, being the maximum fold increase of 1.58 (miR-34a) and the maximum fold decrease of 1.79 (miR-431) (Figure 3). Such a low differential expression (<2-fold) is not expected to have any appreciable biological relevance.[14,15] For each endogenous miRNA that was significantly differentially expressed, target transcripts were assessed *in silico* (Table S1). RNA sequencing (RNA-seq) analysis confirmed that none of the target transcripts showed significant alterations, except for the Retrotransposon-like protein 1 (*RTL1*) transcript. However, the *RTL1* transcript showed the same alteration when compared to AAV5-GFP, indicating that this is rather an AAV5 vector-related effect and not due to miHTT treatment (Table S2).

To ensure that overall expression levels of endogenous miRNAs were not affected, absolute mature miRNA counts were determined in AAV5-miHTT-treated HD71 iPSC-derived neuronal cultures. In total, 4.12×10^7 mature miRNA sequences could be annotated to the human genome, of which 650,793 counts (of 63,914 unique small RNA reads with a maximum of two mismatches to the human genome) were aligned to the mature miHTT sequence (=1.58%) (Figure 4). There are also other endogenous miRNAs expressed at higher levels than miHTT. Thus, the small transcriptome analysis indicates that therapeutic miHTT was expressed at physiological levels, correctly processed, did not influence the expression of endogenous cellular miRNAs, and was unlikely to impact endogenous miRNA gene regulation.

No Altered Expression of *In Silico*-Predicted AAV5-miHTT Off-Target Genes

Although AAV5-miHTT was designed to specifically target the *HTT* mRNA transcript, complementarity with other transcripts might result in off-target lowering of other genes. AAV5-miHTT off-target activity was predicted using BLAST to search for transcripts with partial complementarity with the guide strand using the *Homo sapiens* reference RNA sequence database (refseq_rna GCF_000001405.38 [GRCh38.p12]). The shortest (19 nt), the longest (30 nt), and the most abundant (24 nt) mature miHTT sequences obtained by the small RNA-seq were used for *in silico* prediction using various stringent and less stringent algorithms, accepting up to just 7-nt homology. The off-target activity of the passenger strand was not investigated, as there was no evidence of passenger strand processing after AAV5-miHTT treatment. siSPOTR was used to predict the binding of the AAV5-miHTT seed sequence (nucleotide 2–8) to predominantly the 3′ UTR of target transcripts.[16] From these *in silico* searches, 19 potential off-target genes were identified (10 genes using the BLAST approach, and 9 with the siSPOTR approach) and were assessed by qPCR in RNA samples of AAV5-miHTT-treated cells compared with formulation buffer-treated samples. AAV5-GFP at the same MOI was included as a viral transduction control in order to differentiate between potential off-target effects of miHTT and of AAV5 itself. None of the *in silico*-predicted genes was significantly downregulated or upregulated after AAV5-miHTT treatment or AAV5-GFP treatment of neuronal cell cultures from either HD patient (HD71 of HD180) relative to formulation buffer-treated samples (Figure 5A). There were also no significant changes in mRNA expression of the siSPOTR-predicted off-target genes after AAV5-miHTT or AAV5-GFP treatment (Figure 5B). These data indicate that there are no significant effects of the therapeutic miHTT on non-target genes.

Next-Generation Sequencing Confirmed the Absence of Dysregulated Genes after AAV5-miHTT Treatment

The *in silico* analysis, which was used to predict and study genes that might be affected by AAV5-miHTT, is potentially open to bias, so

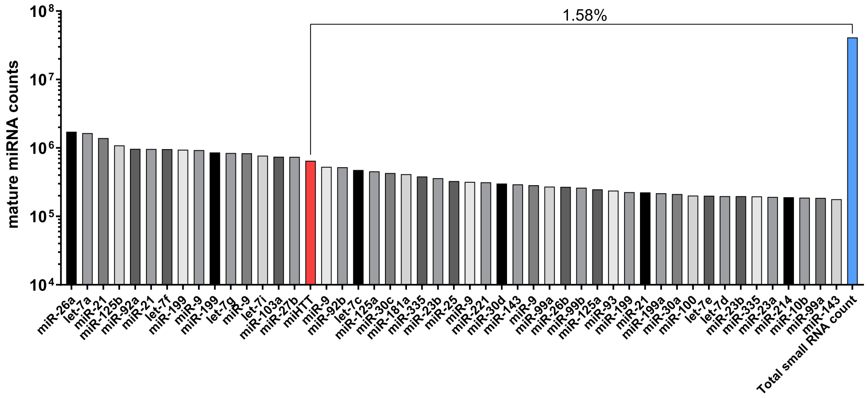

Figure 4. Small RNA Sequencing Analysis of AAV5-miHTT-Treated HD71 iPSC-Derived Neuronal Culture

Top 50 of the most abundant small transcripts. The red bar reflects the number of mature miRNA counts that aligned to miHTT, and the blue bar reflects the total mature small RNA count.

next-generation sequencing (NGS) was performed to examine the dysregulation of genes, either by miHTT or resulting from Htt protein lowering, in an unbiased manner. In total, 60,088 human transcript variants were analyzed using an unbiased RNA-seq approach in HD71 iPSC-derived neuronal cultures treated with AAV5-miHTT at a MOI of 10^5 or 10^7 per well, AAV5-GFP at a MOI of 10^7 (viral transduction control), and formulation buffer (negative control). For NGS, single-end sequence reads were formatted, normalized, clipped, and quality assessed as described in the Materials and Methods section. The total number of reads ranged from 37,734,552 to 60,635,403. Genes that were upregulated or downregulated with a positive or negative fold change of ≥ 1.2 and with a p value <0.05 were considered. For most *in silico*-predicted off-target genes, the changes in gene expression in the high-dose AAV5-miHTT-treated group and viral transduction control group were similar when compared with formulation buffer (Figure 6).

The NGS analysis identified 49 transcripts that were putatively dysregulated by miHTT expression following treatment with AAV5-miHTT (Figure S5). After removing genes that were in low abundance (less than 500 reads) in all treatment groups and transcripts that did not show an AAV5-miHTT dose-dependent response, 12 genes were selected for qPCR validation. Although expression of some genes was lowered after AAV5-miHTT treatment, the AAV5-GFP treatment caused a greater inhibition, indicating a general AAV5 effect rather than a miHTT sequence-specific off-target effect (Figure 7). In addition, the upregulation and downregulation of the transcripts was not consistent between the two cell lines (HD71 and HD180), and in the HD180 cell line the greatest upregulation was observed with AAV5-GFP. Thus, NGS testing in HD71 iPSC-derived neuronal cultures and subsequent qPCR validation of AAV5-miHTT in iPSC-derived neuronal cultures from two different HD patients indicated no evidence of off-target activity.

AAV5-miHTT Does Not Have Off-Target Effects in Human iPSC-Derived Astrocytes

In addition to the neuronal cultures, AAV5 is also known to transduce astrocytes. Therefore, potential off-target activity of AAV5-miHTT, in terms of *in silico*-predicted targets identified by BLAST, siSPOTR, and Ingenuity Pathway Analysis (IPA) was quantified in iPSC-derived astrocytes treated with AAV5-miHTT or AAV5-GFP as a viral transduction control. Human iPSC-derived astrocytes (>99% $GFAP^+$, Figure S6) were transduced with AAV5-miHTT at a MOI of 10^7, AAV5-GFP, or formulation buffer alone as a negative control. One week after transduction, cells were harvested and total RNA was isolated. All *in silico*-predicted off-target genes were shown to be unaffected by AAV5-miHTT treatment, confirming that the lack of sequence-related off-target activity in the neuronal cultures was also observable in human astrocytes (Figure 8).

DISCUSSION

This study builds on data with AAV5-miHTT from a range of animal models including rodents, minipigs, and nonhuman primates by examining human iPSC-derived neuronal cultures and astrocytes from individuals with HD. As in the animal models, AAV5-miHTT was able to dose-dependently transduce human neuronal cultures and induce the expression of mature miHTT. In human iPSC-derived neuronal cultures, AAV5-miHTT treatment was associated with significant reductions in *HTT* mRNA by approximately 57% and Htt protein by 68% with a MOI of 10^7 genome copies (gc). This compares with an approximately 50%–70% reduction in *HTT* mRNA with AAV5-miHTT at 3×10^{13} gc/brain in a minipig HD model and a 98% reduction in mutant Htt protein aggregates in rodent models with AAV5-miHTT at 6.5×10^{10} gc/brain.[5,8] In terms of other similar approaches, VY-HTT01, an AAV1-miRNA gene therapy targeting HTT also under development, has demonstrated the ability to reduce *HTT* mRNA by approximately 70% in the caudate of nonhuman primates (P. Zhou et al., 2018, Hum. Gene Ther., abstract).

A major aim of this study was to investigate the different potential forms of off-target effects by AAV5-miHTT treatment whether directly through binding to non-target mRNA or indirectly through passenger strand activity or perturbation of the endogenous RNAi machinery. Passenger strand activity is a key source of indirect off-target effects for miRNA.[17] AAV5-miHTT uses the miR-451 scaffold that is not targeted by DICER, so processing of this scaffold does not generate a passenger strand. The selection of AAV5-miHTT as a lead therapeutic candidate followed an extensive design and testing program that demonstrated it had strong *HTT* silencing combined with undetectable passenger strand concentration and therefore no passenger strand activity.[4] The lack of passenger strand activity was confirmed in the current study by small RNA sequencing.

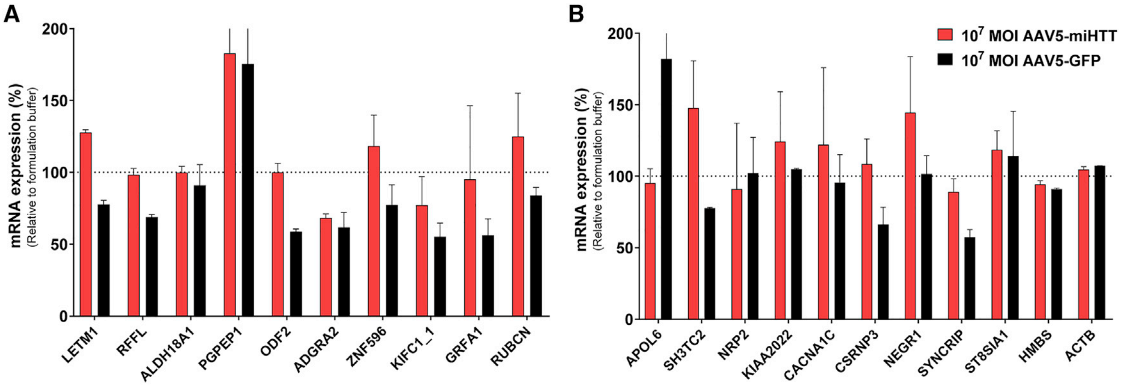

Figure 5. qPCR Analysis of *In Silico*-Predicted AAV5-miHTT Off-Target Genes in HD71 and HD180 Patient iPSC-Derived Neuronal Cultures

(A) BLAST top hits. (B) siSPOTR predicted off-target top list. HD71 and HD180 patient iPSC-derived neuronal cultures were transduced with AAV5-miHTT at a MOI of 10^7 (n = 3), AAV5-GFP at the same MOI (n = 3), or formulation buffer (n = 3). RNA was isolated and qPCR analysis was performed. Genes are normalized against the geometric mean of both housekeeping genes (*ACTB* and *HMBS*). Bars represent average ± SD of the percentage normalized expression with respect to formulation buffer-treated neuronal cultures. Data were evaluated using a paired Student's t test versus formulation buffer control.

Another potential off-target effect of miRNA-based gene therapy is nonspecific perturbation of endogenous gene regulation by saturation of endogenous RNAi machinery.[18,19] This is thought to result from competition between the endogenous and engineered miRNAs for the intracellular factors and pathways that are needed for the processing of primary- and pre-miRNA.[18,19] Additional variables such as promoter strength may affect miRNA expression levels and protein knockdown,[20] and therefore may also perturb endogenous gene regulation. Following transfection of HD iPSC-derived neuronal cultures with AAV5-miHTT, mature miHTT was expressed at physiological levels, accounting for a small proportion (1.58%) of all intracellular miRNA. This indicates that miHTT expression is unlikely to outcompete other endogenous miRNAs and thus is unlikely to influence endogenous RNAi. In separate experimental animal models, however, miRNAs have saturated endogenous pathways, causing fatalities.[18] Another potential form of off-target interference would be if AAV5-miHTT affected the expression of other endogenous miRNA; however, transcriptome analysis indicated that only 10 miRNAs were impacted and the increases/decreases in expression were relatively minor (<2-fold). Also, we showed that the target genes of these minimally differentiated miRNAs were not altered in expression. One of these miRNAs, miR-34a, which is involved in cell cycling, senescence, and apoptosis, has been reported to be downregulated in an HD mouse model;[21] however, there does not appear to be published information on the other miRNAs in HD. Although multiple publications have reported on RNAi as a therapy for HD,[22,23] in some cases, off-target activity has been suggested to be caused by high production of the passenger strand by the miRNA, unrelated from the HTT lowering.[24] We are the first to report (the absence of) changes of cellular miRNAs while significantly lowering mutant HTT mRNA and protein, which we consider an important safety aspect of this therapeutic approach.

To examine whether AAV5-miHTT treatment would impact expression of potential off-target genes, *in silico* analysis was performed using BLAST to search for off-target genes with partial guide strand complementarity and siSPOTR to predict potential off-target effects related to the seed sequence of miHTT. Of the 19 potential off-target genes identified by both *in silico* approaches, none was downregulated after AAV5-miHTT treatment. In some cases, such as the adhesion G protein-coupled receptor A2 (*ADGRA2* gene), which has roles in neural development and the maintenance of the blood-brain barrier,[25] and the kinesin family member C1 (*KIFC1* gene), moderate downregulation was observed with AAV5-miHTT and AAV5-GFP, indicating a potential AAV5-mediated effect. In terms of the clinical impact of potential AAV5-mediated effects, evidence from both pediatric patients and a minipig model showed that administration of the same AAV5 vector directly into the brain was well tolerated, indicating that there is currently no evidence to suggest clinical concerns of AAV5 treatment in humans.[8,26]

In silico analyses have the potential to bias outcomes by selecting the target genes to analyze, and therefore we performed unbiased NGS to confirm the absence of dysregulated genes after AAV5-miHTT treatment in 60,088 distinct human genes. As with the *in silico*-identified targets, the NGS analysis indicated similar upregulation or downregulation with AAV5-miHTT and AAV5-GFP treatment, suggesting an effect due to the AAV5 vector rather than miHTT expression. The regulation of genes did not appear to be consistent between the cell lines from the two HD patients, and the greatest upregulation in the HD180 cell line was observed with AAV5-GFP. This difference could be due to the genetic variation, as well as the individual iPSC generation and subsequent differentiation differences, resulting in slightly different frontal brain-like cell populations. Nevertheless, NGS testing of AAV5-miHTT in iPSC-derived neuronal cultures from two different HD patients indicated no evidence of off-target activity. It would also be interesting to examine those genes in which there was a pronounced difference between AAV5-miHTT and AAV5-GFP, such as the mitochondrial proton/calcium exchanger protein (*LETM1*), apolipoprotein L6 (*APOL6*), and SH3

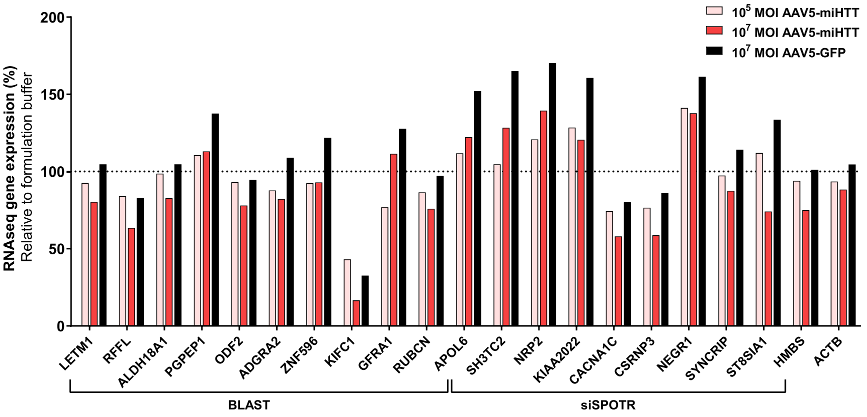

Figure 6. Confirmation of *In Silico*-Predicted AAV5-miHTT Target Genes from RNA-Seq Analysis

HD71 iPSC-derived neuronal cultures were transduced with AAV5-miHTT at a MOI of 10^5 or 10^7, formulation buffer as negative control, and AAV5-GFP at a MOI of 10^7 as viral transduction control (n = 4). RNA was isolated and unbiased RNA sequence analysis was performed. The analysis of the weighted proportions fold change and p value scores between the conditions were calculated using the Baggerley's beta-binomial test. The housekeeping genes *ACTB* and *HMBS* are plotted in the graph to demonstrate equal RNA input between the treatment groups. Data were evaluated using a paired Student's t test versus formulation buffer control.

domain and tetratricopeptide repeats 2 (*SH3TC2*) genes, as these differences may potentially reflect Htt lowering rather than miHTT toxicity.

Given that AAV5 is known to transduce astrocytes in addition to neurons,[6] we analyzed off-target effects of AAV5-miHTT in iPSC-derived astrocytes. AAV5-miHTT treatment of astrocytes had no impact on any of the *in silico*-predicted off-target genes. Therefore, the lack of sequence-related off-target activity observed in neuronal cultures was also seen in astrocytes.

The highest titer we could test, a MOI of 10^7 for AAV5-miHTT, was used on patient iPSC-derived neuronal cultures. To shift this into the perspective of the animal studies, concentrations of vector genome copies per microgram of DNA found back in neuronal cultures were more than 2 logs higher than the highest vector genome concentrations found back in our animal studies, which were performed with the planned clinical doses.[7,8] Therefore, the investigated off-target effects are far beyond the ranges that will be reached in the clinic, giving us a comfortable safety margin. The limitations of higher MOIs were due to a combination of virus concentration, well-to-medium volume, and cell density.

This study confirms that the efficacy of AAV5-miHTT demonstrated in several animal studies also applies to human iPSC-derived neuronal cultures from two individuals with HD. In addition, the study extends the characterization of AAV5-miHTT treatment further by examining potential off-target effects in a cell-, disease-, and species-specific model. The efficacy of neuronal transduction, expression of mature miHTT, reduction of *HTT* mRNA, and suppression of Htt protein with AAV5-miHTT in human neuronal cultures was similar to that observed in animal models for AAV5-miHTT. With the highest MOI of 10^7, the transduction efficiencies were even higher, which fits the experiment to be used for determination of off-target effects. An extensive series of analyses also indicated that AAV5-miHTT treatment was processed correctly without off-target passenger strand and that no cellular miRNAs were dysregulated, indicating that the endogenous RNAi machinery was unaffected by miHTT overexpression. qPCR validation of *in silico*-predicted off-target transcripts, NGS, and pathway analysis confirmed the absence of dysregulated genes due to sequence homology or seed-sequence activity of miHTT. Minor effects on gene expression were observed in both AAV5-miHTT- and AAV5-GFP treated-samples, suggesting that they were due to viral transduction rather than miHTT. Therefore, this wide-ranging analysis indicates the lack of off-target effects associated with AAV5-miHTT treatment and provides confidence in the efficacy and safety profile of this therapeutic candidate as it moves further into clinical studies.

MATERIALS AND METHODS

Cell Culture

HD71 and HD180 neuronal cultures were 2-week-matured frontal brain-like neuronal cells differentiated from ND42229*B iPSCs and CS97iHD-180n2 iPSCs, respectively, by dual inhibition of SMAD signaling.[9] As control cells, ND42245*F iPSCs were differentiated into astrocytes. In all experiments, iPSC-derived frontal brain-like neuronal cultures were seeded in poly(D) and laminin-precoated 24-well plates. For the second coating, laminin solution was aspirated briefly before the cells were seeded to ensure that wells did not dry out. iPSC-derived frontal brain-like neuronal cells were counted before seeding using the NucleoCounter NC-100 (ChemoMetec, Allerod, Denmark). Frontal brain-like neuronal cells were seeded in 1×10^5 cells/well in 0.5 mL of supplemented neuron maturation medium (STEMCELL Technologies, Grenoble, France). Astrocytes were seeded in 5×10^4 cells/well in 0.5 mL of supplemented astrocyte maturation medium (STEMCELL Technologies, Grenoble, France). After seeding, the medium was refreshed twice per week by washing with 1 mL of Dulbecco's PBS (DPBS) before addition of fresh medium.

Viruses and Transduction

AAV5 vector encoding cDNA of the miHTT cassette and enhanced GFP was produced by a baculovirus-based AAV production system (uniQure, Amsterdam, the Netherlands) as described previously.[4] Expression was driven by a combination of the cytomegalovirus early enhancer element and chicken β-actin promoter, and the transcription unit was flanked by two noncoding AAV-derived inverted terminal repeats. Transductions were performed 2 days after the cells

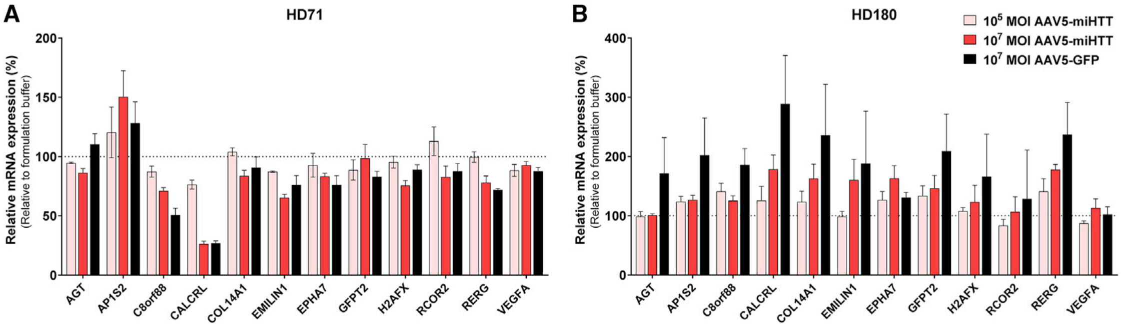

Figure 7. qPCR Analysis of Possible Off-Target Genes in Two Different HD Patient iPSC-Derived Neuronal Cultures

(A) HD71. (B) HD180. HD patient iPSC-derived neuronal cultures were transduced with AAV5-miHTT at a MOI of 10^5 or 10^7, formulation buffer as negative control, and AAV5-GFP at a MOI of 10^7 as viral transduction control (n = 3). RNA was isolated and qPCR analysis was performed. Genes are normalized against the geometric mean of both housekeeping genes (*ACTB* and *HMBS*). Bars represent average ± SD of the percentage normalized expression with respect to formulation buffer-treated neuronal cultures. Data were evaluated using a paired Student's t test versus formulation buffer control.

were seeded using different doses of AAV5-miHTT, AAV5-GFP at a MOI of 10^7, and formulation buffer as negative control (n = 3). Before transduction, the medium was aspirated, and cells were rinsed with 1 mL of DPBS.

Vector Genome Copy Determination

Vector genome copy determination of the transduced cells was performed by first isolating DNA using the DNeasy Blood and Tissue kit protocol (QIAGEN, Venlo, the Netherlands). DNA was eluted in 50 μL of water for injection, and the concentration was measured by NanoDrop 2000 (Thermo Fisher Scientific, Loughborough, UK). Primers specific for the CAG promoter were used to amplify a sequence specific for the transgenes by SYBR Green Fast qPCR (Thermo Fisher Scientific). The vector genome copies per microgam of genomic DNA input of the samples were calculated by interpolation of a standard line of the expression cassette. To define the background levels of the qPCR, a blank sample was subjected to qPCR using the same expression cassette-targeting primers.

RNA Isolation

The medium was aspirated, and the cells washed with PBS before 600 μL of TRIzol (Thermo Fisher Scientific, Loughborough, UK) was added to each well. RNA isolation was performed immediately using the Direct-zol RNA MiniPrep kit (Zymo Research, Irvine, CA, USA) or plates were transferred to −80°C after TRIzol was added and RNA isolated at a later time point. A DNase treatment supplied by the kit was performed during the RNA isolation. RNA concentration was measured using 1 μL in the NanoDrop 2000 (Thermo Fisher Scientific, Loughborough, UK). RT-PCRs were performed on a Biometra TAdvanced thermal cycler (Biometra, Göttingen, Germany).

Random cDNA Synthesis

HTT mRNA lowering was assessed using the DyNAmo cDNA synthesis kit with random hexamer primers (Thermo Fisher Scientific, Loughborough, UK). To determine mRNA expression levels of off-target genes, the Maxima first-strand cDNA synthesis kit was used with random hexamer primers (Thermo Fisher Scientific, catalog no. K1672). For each cDNA synthesis assay, a non-reverse-transcribed control was analyzed. For the off-target genes also a non-DNase-treated sample was taken along as a control.

Mature miHTT Guide Strand-Specific cDNA Synthesis and Guide Strand qPCR

To determine miHTT (miRNA molecule) levels, the TaqMan MicroRNA Reverse Transcription Kit (Thermo Fisher Scientific, Loughborough, UK) and gene-specific RT primers to target miH12-451-23nt and internal control U6 (Thermo Fisher Scientific, Loughborough, UK) were used. Mature miHTT levels in the samples were calculated by normalized control miRNA and relative to the formulation buffer control group. To control for genomic DNA contamination in the samples an RT and a no template control sample were subjected to qPCR using the same primer-probe set specific for miHTT. RT-PCRs were performed on a Biometra TAdvanced thermal cycler (Biometra, Göttingen, Germany).

HTT mRNA qPCR

HTT mRNA levels were quantified by a TaqMan qPCR to assess *HTT* mRNA lowering. TaqMan primer and probe combinations were ordered for the *HTT* (Thermo Fisher Scientific, Loughborough, UK) and *GAPDH* (Thermo Fisher Scientific, Loughborough, UK) genes. For relative expression levels *GAPDH* was used as the housekeeping gene.

Htt Protein Expression

Htt protein expression in cell pellet homogenates was analyzed by an ultrasensitive single molecular counting (SMC) immunoassay (IRBM Science Park, Pomezia, Italy), similar to the previously described analysis of mutant Htt protein.[27] The assay is based on the use of a capture antibody (2B7), coated on magnetic beads, and a detection antibody (MAB2166) labeled with a fluorescent

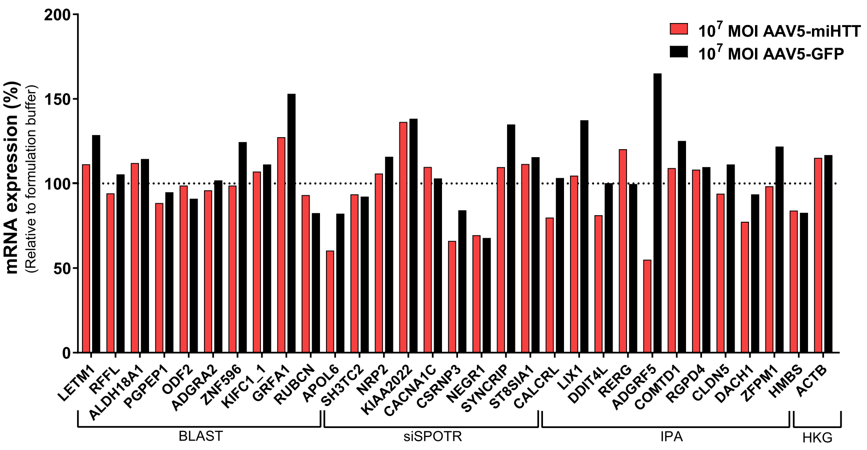

Figure 8. qPCR Analysis of *In Silico*-Predicted Off-Target Genes Analyzed in iPSC-Derived Astrocytes

iPSC-derived astrocytes were transduced with AAV5-miHTT at a MOI of 10^7 (n = 3), AAV5-GFP at a MOI of 10^7 (n = 3), and formulation buffer (n = 3). RNA was isolated and qPCR analysis was performed. Data were evaluated using a paired Student's t test versus formulation buffer control. HKG, housekeeping genes.

dye. The analyte is quantified with respect to standard curve of recombinant Htt (N548 Q23 recombinant Htt protein). Htt protein concentrations were expressed in pg Htt/μg total protein and then calculated as percentage of expression with respect to the control (PBS-treated) group.

Off-Target Genes qPCR

SYBR Green qPCRs were performed to test AAV5-miHTT off-target effects, using primers designed based on the sequence of the human transcript (https://www.ensembl.org). The primers were tested in six different qPCR runs. For sequences, see Table S3.

NGS Sequencing and Analysis

500–1000 ng of total RNA from the HD71 frontal brain-like neuronal cells transduced with formulation buffer, AAV5-miHTT, or AAV5-GFP was reverse transcribed and single-end sequence reads were generated using the Illumina HiSeq2500 system (BaseClear, Leiden, the Netherlands). FASTQ sequence files were generated using the Illumina Casava pipeline version 1.8.3. Initial quality assessment was based on data passing the Illumina chastity filtering. Subsequently, reads containing PhiX control signal were removed using an in-house filtering protocol. In addition, reads containing (partial) adapters were clipped (up to a minimum read length of 50 bp). The second quality assessment was based on the remaining reads using the FASTQC quality control tool version 0.10.0. Each condition was analyzed in quadruplicates. The number of reads varied from 39,153,379 to 60,635,403, with high average quality scores of 38.30 to 38.53.

The analysis of the weighted proportions fold change and p value scores between the conditions were calculated using Baggerley's beta-binomial test. The fold change in gene expression was calculated for each comparison. The weighted proportions fold change and p value scores were uploaded in IPA software (version 01-13). With IPA, genomics data can be analyzed and relationships between genes can be visualized. In IPA a selection was done on the genes in which the following cut-offs and settings were used: genes that were upregulated or downregulated with a positive or negative fold change of R1.2 and with a p value <0.05 were considered. The fold change cut-off was set on 1.2 since a lower fold change is within normal variability. Comparisons between the AAV5-miHTT, formulation buffer, and AAV5-GFP treatment groups were performed. The fold change and p value per transcript per comparison were loaded into IPA to identify direct and indirect off-targets or relationships.

Small RNA Sequencing

Total RNA of HD71 iPSC-derived frontal brain-like neuronal culture transduced with AAV5-miHTT at a MOI of 10^7 and formulation buffer was sent out for small RNA sequencing (BaseClear, Leiden, the Netherlands). Each condition was analyzed in quadruplicate. Small RNA sequencing libraries for the Illumina platform were prepared and sequenced at BaseClear (Leiden, the Netherlands). Total RNA was first assessed for quality by measuring the RNA integrity number (RIN) value on a Bioanalyzer 2100 (Agilent, Santa Clara, CA, USA) with the RNA 6000 Nano kit (Agilent, Santa Clara, CA, USA), analyzed using the 2100 Expert software (version B.02.09.SI725) and used as input for library preparation using the NEXTflex small RNA-seq kit v3 (Bioo Scientific, Austin, TX, USA). Briefly, the small RNA species were subjected to ligation with 3′ and 5′ RNA adapters, first-strand reverse transcription, and PCR amplification with sample-specific barcodes. The PCR products were separated on Tris-borate-EDTA (TBE)-PAGE electrophoresis, and the expected band around 140–160 bp was recovered for each sample. The resulting sequencing libraries were checked and quantified on a BioAnalyzer (Agilent, Santa Clara, CA, USA). Next, the libraries were multiplexed, clustered, and sequenced on an Illumina HiSeq 2500 with a single-read 50 cycles sequencing protocol plus indexing. The sequencing run was analyzed with the Illumina CASAVA pipeline (v1.8.3), with demultiplexing based on sample-specific barcodes. The raw sequencing data produced were processed by removing the sequence reads that were of too low quality (only "passing filter" reads were selected) and discarding reads that aligned against the PhiX control library. In addition, reads containing (partial) Illumina adapters were clipped (up to a minimum read length of 50 bp). The second quality assessment was based on the remaining reads using the FASTQC quality control tool version 0.10.0. The number of reads varied from 14,531,779 to 17,758,794, with high average quality scores of 38.49 to 38.61. The analysis of the miRNA processing of AAV5-miHTT was performed using CLC Genomics Workbench 10 (version 10.1.1).

In Silico Off-Target Prediction

AAV5-miHTT off-target effects were assessed using the NCBI BLAST tool (https://blast.ncbi.nlm.nih.gov/Blast.cgi?PAGE_TYPE=BlastSearch) using different parameters to ensure all possible off-target effects were identified. Off-target effects were checked for the shortest (19 nt), the longest (29 nt), and most abundant (24 nt) miHTT variants. The analysis was performed using two different databases (Reference RNA sequences [refseq_rna] and Human genomic plus transcript [Human G+T]), using both a stringent, highly similar sequences (megablast) algorithm and a less stringent, somewhat similar sequences (blastn) algorithm to check for off-target effects. For all three miHTT variants, the top 100 hits from all four analyses, two databases, and for each a stringent and less-stringent algorithm were used to generate a list of individual genes. The siSPOTR tool (https://sispotr.icts.uiowa.edu/) was used to assess the off-target effects of the seed sequence of AAV5-miHTT, which is ranked by a probability of off-target score (POTS) and transcript POTS (tPOTS) (see Supplemental Materials and Methods).[16] From the full list of genes from the siSPOTR analysis we selected the top 15 genes in the off-target analysis (Table S4).

SUPPLEMENTAL INFORMATION

Supplemental Information can be found online at https://doi.org/10.1016/j.omtm.2019.09.010.

AUTHOR CONTRIBUTIONS

M.M.E. designed and conducted the experiments, M.M.E. and S.K. analyzed the data and wrote the paper. S.K., C.C.B., and M.S.-G. conducted the experiments, S.K., R.M., J.A.D., and A.V. analyzed the data, and S.J.v.D., P.K., and M.M.E. supervised the study.

CONFLICTS OF INTEREST

All author are employees and shareholders of uniQure B.V.

ACKNOWLEDGMENTS

This work was carried out and funded by uniQure biopharma B.V. The authors thank Ellen Broug, Joseph Higgins, and Eileen Sawyer for critically reviewing the manuscript and Mike Lappin from GK Pharmacomm Ltd. for help in drafting the manuscript.

REFERENCES

1. Rawlins, M.D., Wexler, N.S., Wexler, A.R., Tabrizi, S.J., Douglas, I., Evans, S.J., and Smeeth, L. (2016). The prevalence of Huntington's disease. Neuroepidemiology *46*, 144–153.
2. Caron, N.S, Wright, G.E.B., and Hayden, M.R. (1998). Huntington disease. In GeneReviews [Internet], M.P. Adam, H.H. Ardinger, and R.A. Pagon, et al., eds. (University of Washington), https://www.ncbi.nlm.nih.gov/books/NBK1305/.
3. Saudou, F., and Humbert, S. (2016). The biology of huntingtin. Neuron *89*, 910–926.
4. Miniarikova, J., Zanella, I., Huseinovic, A., van der Zon, T., Hanemaaijer, E., Martier, R., Koornneef, A., Southwell, A.L., Hayden, M.R., van Deventer, S.J., et al. (2016). Design, characterization, and lead selection of therapeutic miRNAs targeting huntingtin for development of gene therapy for Huntington's disease. Mol. Ther. Nucleic Acids *5*, e297.
5. Miniarikova, J., Zimmer, V., Martier, R., Brouwers, C.C., Pythoud, C., Richetin, K., Rey, M., Lubelski, J., Evers, M.M., van Deventer, S.J., et al. (2017). AAV5-miHTT gene therapy demonstrates suppression of mutant huntingtin aggregation and neuronal dysfunction in a rat model of Huntington's disease. Gene Ther. *24*, 630–639.
6. Samaranch, L., Blits, B., San Sebastian, W., Hadaczek, P., Bringas, J., Sudhakar, V., Macayan, M., Pivirotto, P.J., Petry, H., and Bankiewicz, K.S. (2017). MR-guided parenchymal delivery of adeno-associated viral vector serotype 5 in non-human primate brain. Gene Ther. *24*, 253–261.
7. Spronck, E.A., Brouwers, C.C., Vallès, A., de Haan, M., Petry, H., van Deventer, S.J., Konstantinova, P., and Evers M, M. (2019). AAV5-miHTT Gene Therapy Demonstrates Sustained Huntingtin Lowering and Functional Improvement in Huntington Disease Mouse Models. Mol. Ther. Methods Clin. Dev. *16*, 334–343.
8. Evers, M.M., Miniarikova, J., Juhas, S., Vallès, A., Bohuslavova, B., Juhasova, J., Skalnikova, H.K., Vodicka, P., Valekova, I., Brouwers, C., et al. (2018). AAV5-miHTT gene therapy demonstrates broad distribution and strong human mutant huntingtin lowering in a Huntington's disease minipig model. Mol. Ther. *26*, 2163–2177.
9. Chambers, S.M., Fasano, C.A., Papapetrou, E.P., Tomishima, M., Sadelain, M., and Studer, L. (2009). Highly efficient neural conversion of human ES and iPS cells by dual inhibition of SMAD signaling. Nat. Biotechnol. *27*, 275–280.
10. Cheloufi, S., Dos Santos, C.O., Chong, M.M., and Hannon, G.J. (2010). A dicer-independent miRNA biogenesis pathway that requires Ago catalysis. Nature *465*, 584–589.
11. Cifuentes, D., Xue, H., Taylor, D.W., Patnode, H., Mishima, Y., Cheloufi, S., Ma, E., Mane, S., Hannon, G.J., Lawson, N.D., et al. (2010). A novel miRNA processing pathway independent of Dicer requires Argonaute2 catalytic activity. Science *328*, 1694–1698.
12. Herrera-Carrillo, E., and Berkhout, B. (2017). Dicer-independent processing of small RNA duplexes: mechanistic insights and applications. Nucleic Acids Res. *45*, 10369–10379.
13. Yoda, M., Cifuentes, D., Izumi, N., Sakaguchi, Y., Suzuki, T., Giraldez, A.J., and Tomari, Y. (2013). Poly(A)-specific ribonuclease mediates 3′-end trimming of Argonaute2-cleaved precursor microRNAs. Cell Rep. *5*, 715–726.
14. Han, M.S., Lee, J.M., Kim, S.N., Kim, J.H., and Kim, H.S. (2018). Human papillomavirus 16 oncoproteins downregulate the expression of miR-148a-3p, miR-190a-5p, and miR-199b-5p in cervical cancer. BioMed Res. Int. *2018*, 1942867.
15. Chen, Y., Zhao, B., Liu, M., Wang, J., Qiu, X., Zhu, C., and Wu, X. (2018). MicroRNAs profiling identifies miR-125a and its target gene Wnt2 in skins of different haired rabbits. Front. Genet. *9*, 628.
16. Boudreau, R.L., Spengler, R.M., Hylock, R.H., Kusenda, B.J., Davis, H.A., Eichmann, D.A., and Davidson, B.L. (2013). siSPOTR: a tool for designing highly specific and potent siRNAs for human and mouse. Nucleic Acids Res. *41*, e9.
17. Søkilde, R., Newie, I., Persson, H., Borg, Å., and Rovira, C. (2015). Passenger strand loading in overexpression experiments using microRNA mimics. RNA Biol. *12*, 787–791.
18. Grimm, D., Streetz, K.L., Jopling, C.L., Storm, T.A., Pandey, K., Davis, C.R., Marion, P., Salazar, F., and Kay, M.A. (2006). Fatality in mice due to oversaturation of cellular microRNA/short hairpin RNA pathways. Nature *441*, 537–541.
19. Khan, A.A., Betel, D., Miller, M.L., Sander, C., Leslie, C.S., and Marks, D.S. (2009). Transfection of small RNAs globally perturbs gene regulation by endogenous microRNAs. Nat. Biotechnol. *27*, 549–555.
20. Lebbink, R.J., Lowe, M., Chan, T., Khine, H., Wang, X., and McManus, M.T. (2011). Polymerase II promoter strength determines efficacy of microRNA adapted shRNAs. PLoS ONE *6*, e26213.
21. Reynolds, R.H., Petersen, M.H., Willert, C.W., Heinrich, M., Nymann, N., Dall, M., Treebak, J.T., Björkqvist, M., Silahtaroglu, A., Hasholt, L., and Nørremølle, A. (2018). Perturbations in the p53/miR-34a/SIRT1 pathway in the R6/2 Huntington's disease model. Mol. Cell. Neurosci. *88*, 118–129.
22. Stanek, L.M., Sardi, S.P., Mastis, B., Richards, A.R., Treleaven, C.M., Taksir, T., Misra, K., Cheng, S.H., and Shihabuddin, L.S. (2014). Silencing mutant huntingtin by adeno-associated virus-mediated RNA interference ameliorates disease manifestations in the YAC128 mouse model of Huntington's disease. Hum. Gene Ther. *25*, 461–474.

23. Ochaba, J., Monteys, A.M., O'Rourke, J.G., Reidling, J.C., Steffan, J.S., Davidson, B.L., and Thompson, L.M. (2016). PIAS1 regulates mutant Huntingtin accumulation and Huntington's disease-associated phenotypes in vivo. Neuron *90*, 507–520.

24. McBride, J.L., Boudreau, R.L., Harper, S.Q., Staber, P.D., Monteys, A.M., Martins, I., Gilmore, B.L., Burstein, H., Peluso, R.W., Polisky, B., et al. (2008). Artificial miRNAs mitigate shRNA-mediated toxicity in the brain: implications for the therapeutic development of RNAi. Proc. Natl. Acad. Sci. USA *105*, 5868–5873.

25. UniProt (2019). UniProtKB - Q96PE1 (AGRA2_HUMAN), https://www.uniprot.org/uniprot/Q96PE1.

26. Tardieu, M., Zérah, M., Gougeon, M.L., Ausseil, J., de Bournonville, S., Husson, B., Zafeiriou, D., Parenti, G., Bourget, P., Poirier, B., et al. (2017). Intracerebral gene therapy in children with mucopolysaccharidosis type IIIB syndrome: an uncontrolled phase 1/2 clinical trial. Lancet Neurol. *16*, 712–720.

27. Fodale, V., Boggio, R., Daldin, M., Cariulo, C., Spiezia, M.C., Byrne, L.M., Leavitt, B.R., Wild, E.J., Macdonald, D., Weiss, A., and Bresciani, A. (2017). Validation of ultrasensitive mutant huntingtin detection in human cerebrospinal fluid by single molecule counting immunoassay. J. Huntingtons Dis. *6*, 349–361.

OMTM, Volume *15*

Supplemental Information

AAV5-miHTT Lowers Huntingtin mRNA and Protein without Off-Target Effects in Patient-Derived Neuronal Cultures and Astrocytes

Sonay Keskin, Cynthia C. Brouwers, Marina Sogorb-Gonzalez, Raygene Martier, Josse A. Depla, Astrid Vallès, Sander J. van Deventer, Pavlina Konstantinova, and Melvin M. Evers

Supplementary Figure 1. Schematic of neuronal differentiation of induced-pluripotent stem cells (iPSC). To acquire HD patient-derived neuronal cultures, fibroblasts from HD patients with 71 CAG repeats (HD71, shown) or 180 CAG repeats (HD180, not shown) were reprogrammed to iPSC and subsequently differentiated into frontal brain-like neuronal cells. iPSC colonies were isolated and induced to neural progenitor cells by rosette isolation and neural tube formation. Neural tubes were selected and differentiated to neuronal precursors. Neuronal precursors were matured for 2 weeks.

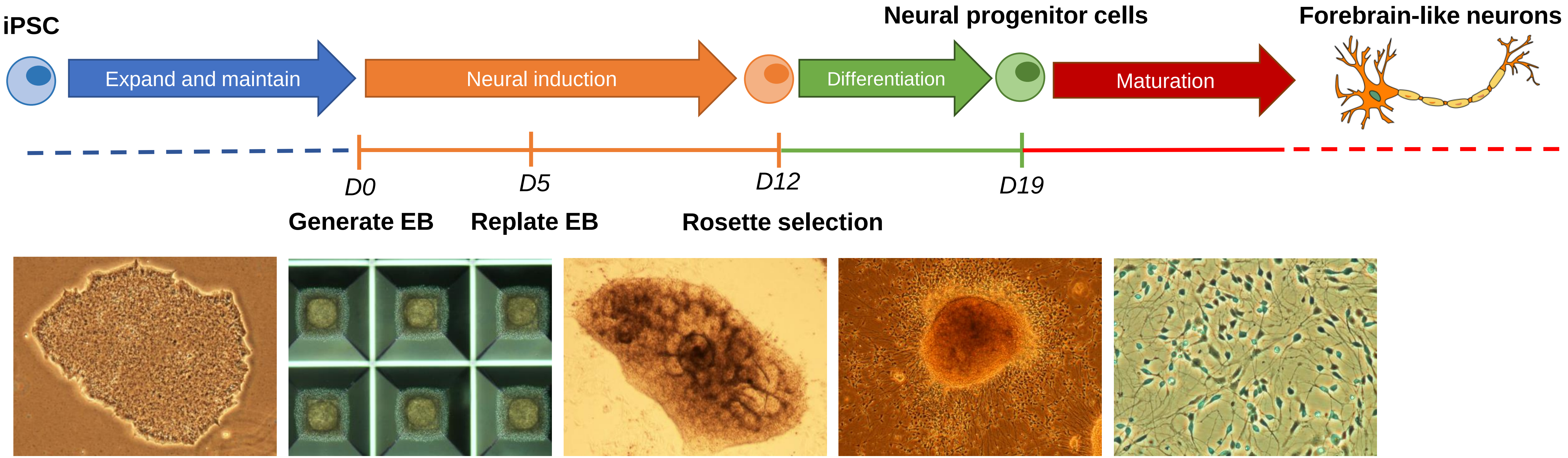

Supplementary Figure 2. Characterization of neuronal population. Characterization of HD iPSC-derived neuronal cultures by immunohistochemistry with antibodies detecting MAP2 and GFAP. A, E) DAPI staining for cell nuclei. B, F) MAP2 staining for neuronal cells. C, G) GFAP staining for astrocytes. D, H) Merged image of DAPI, MAP2 and GFAP staining showed the neuronal population is a mixture of neurons and astrocytes.

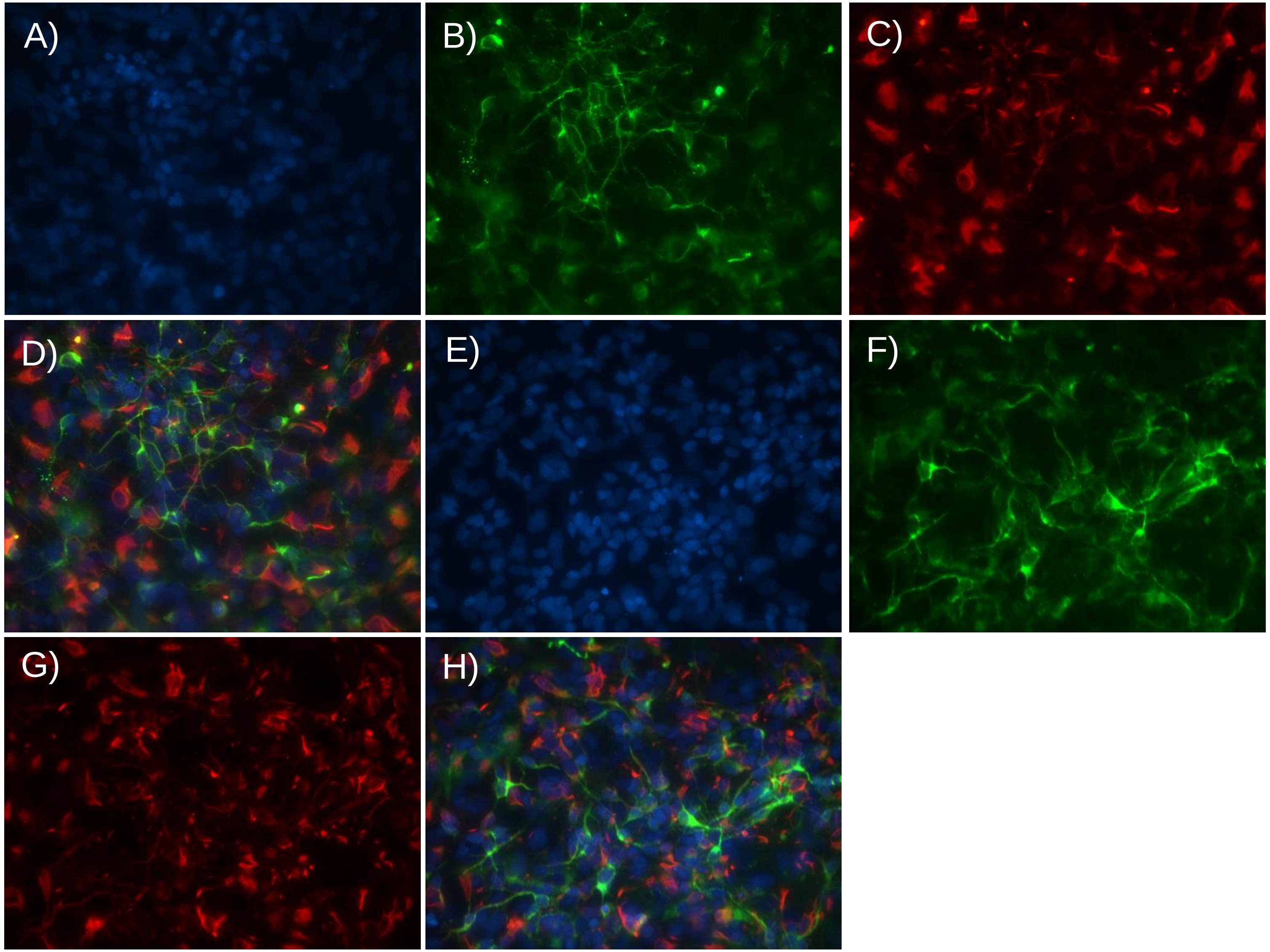

Supplementary Figure 3. Neuronal morphology of HD71 iPSC-derived neuronal cultures after 10 days of transduction. HD patient iPSC-derived neuronal culture treated with AAV5-miHTT MOI 10^5, MOI 10^7, AAV5-GFP MOI 10^7 and formulation buffer as control (see Supplementary Figure 3). Cells are kept in culture for 10 days after transduction and medium was refreshed twice a week. T=D0) pictures of neuronal culture before transduction. T=D4) pictures of neuronal culture taken 4 days after transduction. T=D7) pictures of neuronal culture taken 7 days after transduction. T=D10) pictures of neuronal culture taken 10 days after transduction.

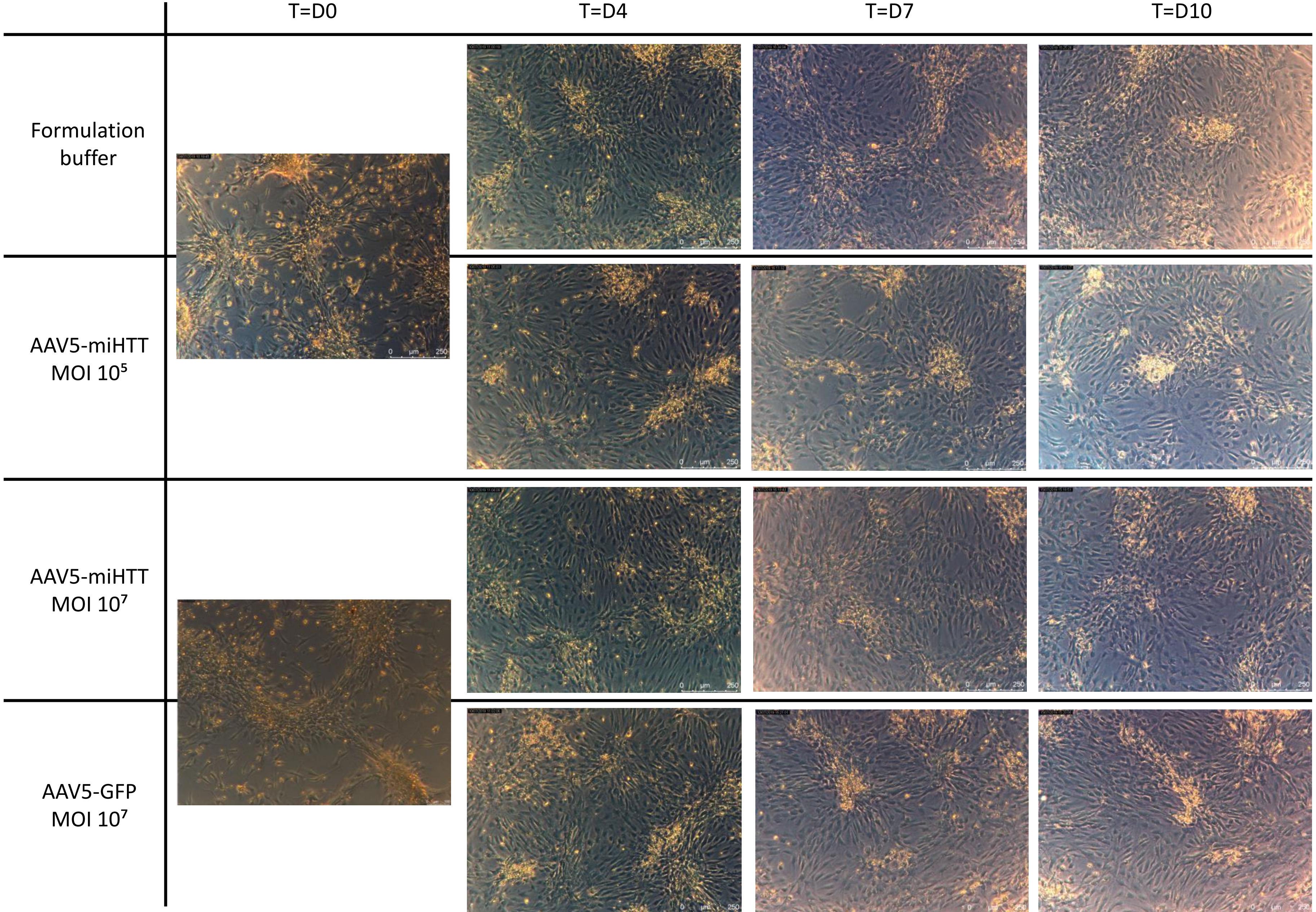

Supplementary Figure 4. Small RNA sequencing analysis of miHTT processing in HD patient iPSC-derived neuronal culture. Most abundant mature miHTT guide strands with sequence distribution in %. The *in-silico* predicted guide strand was 22 nucleotides in length.

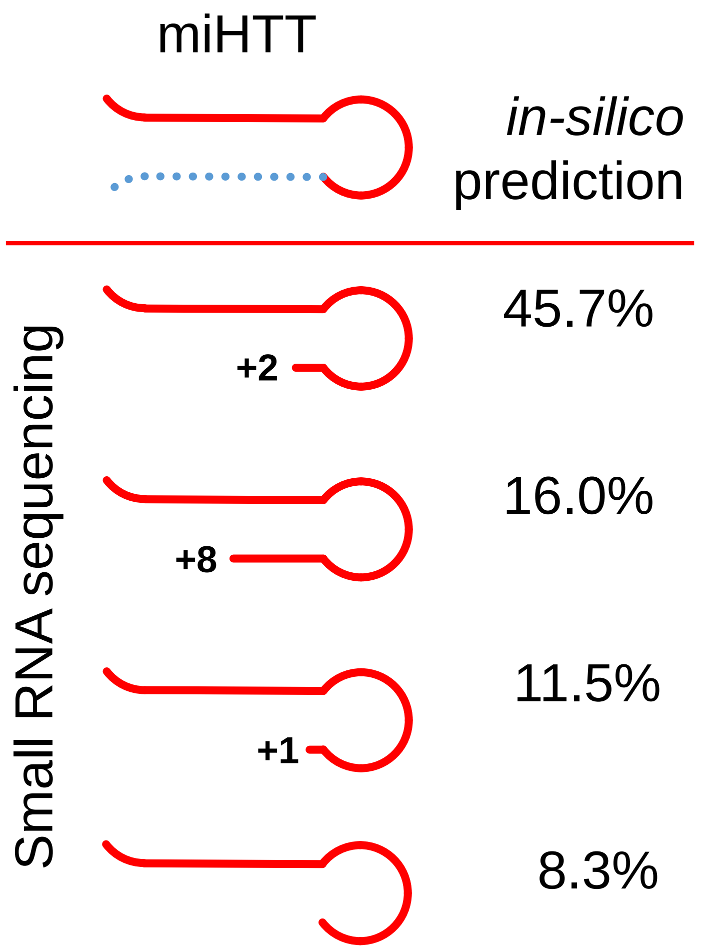

Supplementary Figure 5. Venn diagrams of RNA sequence comparisons of treatment groups. To investigate miHTT-related total transcriptome changes and gene pathway changes, Ingenuity Pathway Analysis (IPA) was performed to compare differentially expressed genes following AAV5-miHTT, AAV5-GFP and formulation buffer treatment.

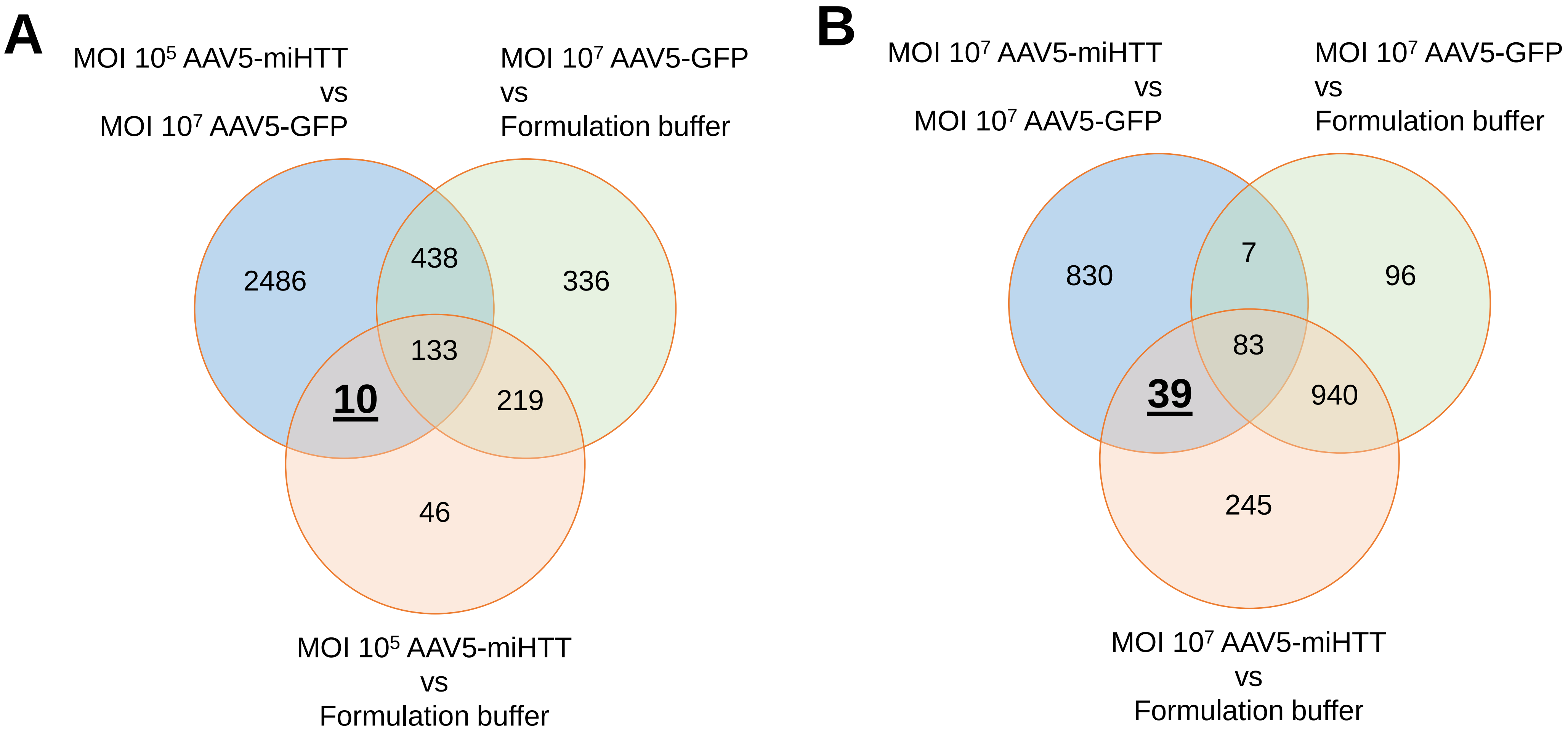

Supplementary Figure 6. Characterization of mature iPSC-derived astrocytes. Mature iPSC-derived astrocytes were fixed and stained for β tubulin III, tyrosine hydroxylase and GFAP. A, D, G) DAPI staining of astrocytes. B) Astrocytes were negative for β tubulin III. C) Merged image of DAPI and β tubulin III. Cells were negative for β tubulin III in all cells. E) Astrocytes were negative for tyrosine hydroxylase. F) Merged image of DAPI and tyrosine hydroxylase showed that zero cells were positive for tyrosine hydroxylase. H) Astrocytes were GFAP+. I) Merged image of DAPI and GFAP showed that all cells are positive for GFAP.

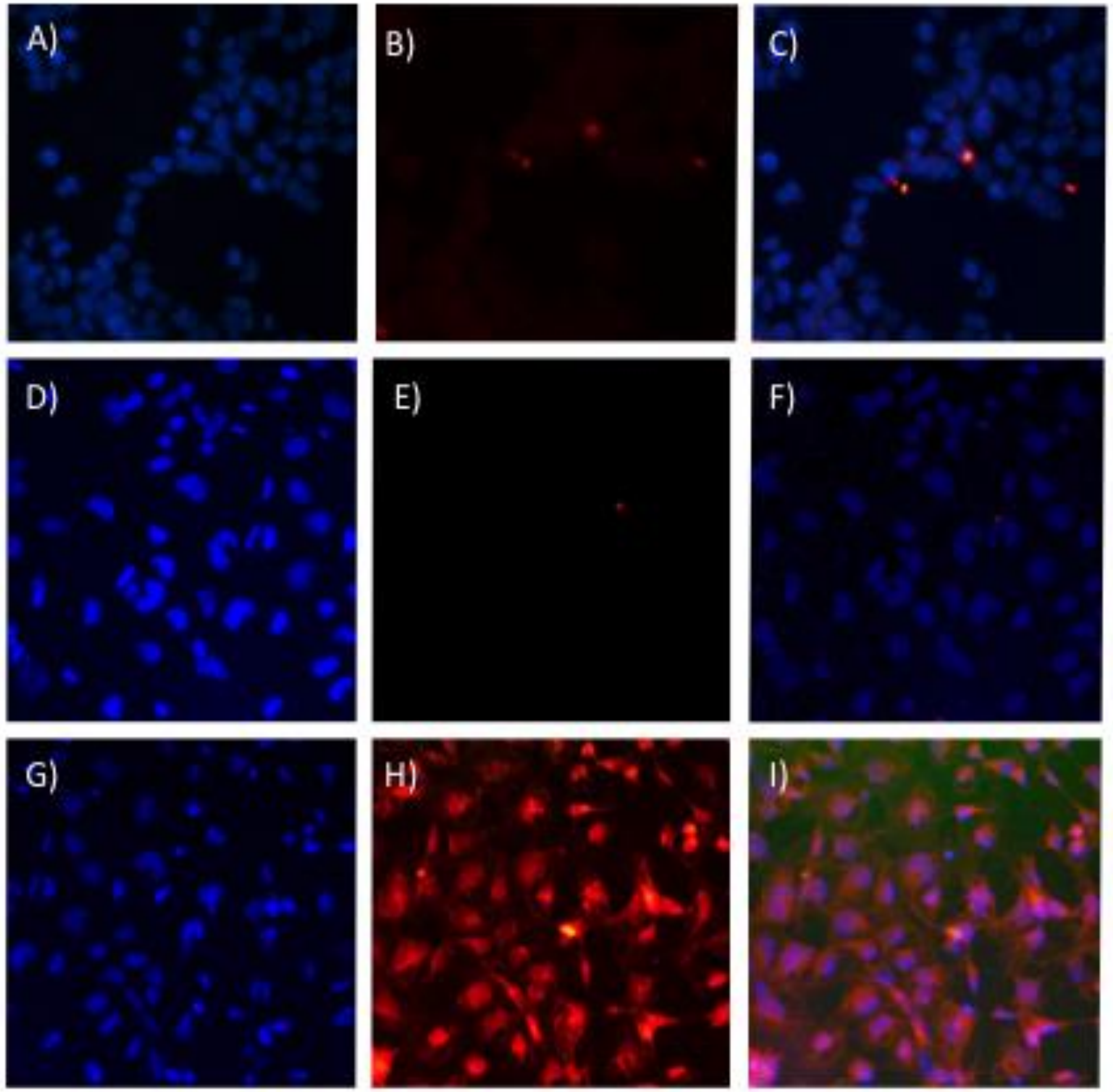

Supplementary Table 1. Target genes of the 10 differentially expressed miRNAs. For the 10 differentially expressed miRNAs the target genes are shown according to BLAST and siSPOTR.

miRNA	miRbase naming	Sequence miRbase	Mature reads	Target gene BLAST	Target gene siSPOTR
Let-7C	hsa-let-7c-5p MIMAT0000064	5'- UGAGGUAGUAGGUUGUAUGGUU -3'	6,84E+07	*HMGCR*	*IGF2BP1*
Mir-26a-1	hsa-miR-26a-5p MIMAT0000082	5'- UUCAAGUAAUCCAGGAUAGGCU -3'	2,85E+07	*CHST9*	*POLH*
Mir-26a-2	hsa-miR-26a-5p MIMAT0000082	5'- UUCAAGUAAUCCAGGAUAGGCU -3'	2,85E+07	*CHST9*	*POLH*
Mir-34a	hsa-miR-34a-5p MIMAT0000255	5'- UGGCAGUGUCUUAGCUGGUUGU -3'	1,56E+05	*SLC13A1*	*HCN3*
Mir-199a-1	hsa-miR-199a-5p MIMAT0000231	5'- CCCAGUGUUCAGACUACCUGUUC -3'	1,83E+06	*TRIP11*	*ZNF763*
Mir-199a-2	hsa-miR-199a-5p MIMAT0000231	5'- CCCAGUGUUCAGACUACCUGUUC -3'	1,83E+06	*TRIP11*	*ZNF763*
Mir-199b	hsa-miR-199b-5p MIMAT0000263	5'- CCCAGUGUUUAGACUAUCUGUUC -3'	7,08E+05	*TRIP11*	*ZNF763*
Mir-431	hsa-miR-431-5p MIMAT0001625	5'- UGUCUUGCAGGCCGUCAUGCA -3'	2,24E+02	*RTL1*	*PLEKHG4B*
Mir-450b	hsa-miR-450b-5p MIMAT0004909	5'- UUUUGCAAUAUGUUCCUGAAUA -3'	1,29E+04	*TM2D3*	*RBMS3*
Mir-503	hsa-miR-503-5p MIMAT0002874	5'- UAGCAGCGGGAACAGUUCUGCAG -3'	5,95E+04	*FOXK1*	*RASEF*

Supplementary Table 2. Transcript expressions in fold change compared to formulation buffer, after AAV5-miHTT and AAV5-GFP treatment, with corresponding P-values. For all target genes we have assessed the transcript expression in HD71 patient iPSC-derived neuronal cultures by RNAseq. Shown are the weighted proportions fold changes in transcript expression after transduction with AAV5-miHTT MOI 10^7 and AAV5-GFP MOI 10^7 compared to formulation buffer treatment, calculated by Baggerley's test. Also, the P-values are shown, calculated by Baggerley's test.

		AAV5-miHTT MOI 10^7 vs Formulation buffer		AAV5-GFP MOI 10^7 vs Formulation buffer	
	**Target gene**	**Fold change**	**P-value**	**Fold change**	**P-value**
**BLAST**	*HMGCR*	-1,2429	0,2188	-1,1564	0,4061
	CHST9	1,8327	0,5325	2,0164	0,4642
	SLC13A1	1	0,9310	1	0,9310
	TRIP11	1,0445	0,8973	1,0847	0,8094
	RTL1	**-2,8416**	**0,0001**	**-2,2398**	**0,0015**
	TM2D3	-1,0941	0,6772	-1,0492	0,8236
	FOXK1	-1,0226	0,9750	-1,0772	0,9185
**siSPOTR**	*IGF2BP1*	-1,0201	0,9347	-1,0333	0,8941
	POLH	2,4418	0,0272	2,2163	0,0565
	HCN3_1	-1,5359	0,7659	-1,2696	0,8630
	HCN3_2	-1,5004	0,7344	-1,6231	0,6934
	ZNF763	-1,0689	0,9862	1,7740	0,8636
	PLEKHG4B	-1,0017	0,9986	-1,0117	0,9909
	RBMS3	-1,4105	0,4023	-1,2376	0,5941
	RASEF	-1,7804	0,9187	-1,0322	0,9949

Supplementary Table 3. SYBR Green qPCR primer sequences

Gene	ENSEMBL transcript #	Fw Primer name	Sequence (5' - 3')	Length	Tm	GC%	Rv primer name	Sequence (5' - 3')	Length	Tm	GC%	Product length (bp)
LETM1	ENST00000302787.2	hLETM1_Fw1	ATGGATCGACACCAAGATCG	20	60,89	50,00	hLETM1_Rv1	ACCACCACGAACACAAGGA	19	59,99	52,63	141
RFFL	ENST00000315249.11	hRFFL_Fw2	CAACCTCCCCTCTTCATCTG	20	59,65	55,00	hRFFL_Rv2	AGACGGGTTCCTCTTGATCC	20	60,46	55,00	107
ALDH18A1	ENST00000371224.6	hALDH18A1_Fw1	AAGCATGCCAAGAGAATCGT	20	59,84	45,00	hALDH18A1_Rv1	CACCAGCATCATCTCTCTGC	20	59,53	55,00	144
PGPEP1	ENST00000269919.10	hPGPEP1_Fw1	GTGGGAGAAGCACAGTCCAC	20	60,72	60,00	hPGPEP1_Rv1	TGTAGCCCTTGTTGTGTCCA	20	60,15	50,00	146
ODF2	ENST00000351030.7	hODF2_Fw1	CCCCCTGGAAAATCATCTG	19	60,26	52,63	hODF2_Rv1	TAACCGCATCACTGAGACCA	20	60,26	50,00	111
ADGRA2	ENST00000412232.2	hADGRA2_Fw1	CCCGGCCTTCTGCCTAAC	18	62,99	66,67	hADGRA2_Rv1	AGTGACAGTCCCAGGAAGGA	20	59,68	55,00	92
ZNF596	ENST00000308811.8	hZNF596_Fw1	TTCCCAATTGGAGCAAGTAGA	21	59,69	42,86	hZNF596_Rv1	GTGGACGTGCCCTTCTGATA	20	61,07	55,00	126
KIFC1_1	ENST00000428849.6	hKIFC1_1_Fw1	TGCAACGACCAAAATTACCA	20	59,97	40,00	hKIFC1_1_Rv1	CTGTGGCAATAGCTGTGGAA	20	59,86	50,00	134
GFRA1	ENST00000369236.5	hGFRA1_Fw1	TGGAGGATTCCCCATATGAA	20	60,09	45,00	hGFRA1_Rv1	TTGTTCCCTTTGGGAATGTG	20	60,72	45,00	97
RUBCN	ENST00000273582.9	hRUBCN_Fw1	CTGGAAGCAGTGGAACAGAA	20	59,01	50,00	hRUBCN_Rv1	CTGTCAGGCTCTGGCTCTTT	30	59,75	55,00	112
APOL6	ENST00000409652.4	hAPOL6_Fw1	ACACAGATTTGCTGCCACAG	20	59,90	50,00	hAPOL6_Rv1	GCTCCACGTCTTCACACAGA	20	60,03	55,00	114
SH3TC2	ENST00000515425.5	hSH3TC2_Fw1	TCCTTCCAAGGATCCAACTG	20	60,04	50,00	hSH3TC2_Rv1	GCGGCTCTTTACACAGAAGG	20	60,02	55,00	121
NRP2	ENST00000360409.7	hNRP2_Fw1	ACTACCCCTCCCACCAGAAC	20	60,23	60,00	hNRP2_Rv1	CCATCCCGAATCTCGATAAA	20	59,86	45,00	136
KIAA2022	ENST00000055682.10	hKIAA2022_Fw1	CAGCCAACGGAGAAAACACT	20	60,29	50,00	hKIAA2022_Rv1	CGGTGTAGGCTGGATAGGTG	20	60,53	60,00	119
CACNA1C	ENST00000399655.5	hCACNA1C_Fw1	CTGCATCAGCATTGTCGAAT	20	59,83	45,00	hCACNA1C_Rv1	GATCGCTAAGGCCACACAAT	20	60,10	50,00	82
CSRNP3	ENST00000342316.8	hCSRNP3_Fw1	AGTGGGGACAGTGTCAATCC	20	59,82	60,25	hCSRNP3_Rv1	CCTTGCCTCCTGGTGAAGTA	20	60,25	55,00	134
NEGR1	ENST00000357731.9	hNEGR1_Fw1	GATGGTCAGAAAAGGGGACA	20	59,90	50,00	hNEGR1_Rv1	CTTATCACCTCCCGCAAAAA	20	60,07	45,00	109
SYNCRIP	ENST00000369622.7	hSYNCRIP_Fw1	AGGACCAGATGAGGCAAAAA	20	59,67	45,00	hSYNCRIP_Rv1	AACAGAAGGCTGCTGACCTG	20	60,59	55,00	130
ST8SIA1	ENST00000396037.8	hST8SIA1_Fw1	TACATCTTCCCCGTCTACCG	20	59,95	55,00	hST8SIA1_Rv1	GTCGCAGCAGTCTTCCATTT	20	60,41	50,00	132

Gene	ENSEMBL transcript #	Fw Primer name	Sequence (5' - 3')	Length	Tm	GC%	Rv primer name	Sequence (5' - 3')	Length	Tm	GC%	Product length (bp)
CALCRL	ENST00000392370.7	hCALCRL_Fw1	TGGACACGGATTGTCTATTGC	21	60,91	47,62	hCALCRL_Rv1	AATCCTTTGGCAACTTAGGC	20	58,33	45	82
LIX1	ENST00000274382.8	hLIX1_Fw1	GCCTCCCTTTGTGAGTTACG	20	59,73	55	hLIX1_Rv1	GATCAGGGCCACTTTAGCTG	20	59,84	55	112
DDIT4L	ENST00000273990.6	hDDIT4L_Fw1	TGCTGGACTGTGGCTATCAC	20	59,86	55	hDDIT4L_Rv1	GAGGTTGGGTTCAGGAACAA	20	59,94	50	80
RERG	ENST00000256953.6	hRERG_Fw1	GATTTCTGACCAAACGGTTCA	21	59,96	42,86	hRERG_Rv1	ATCATCGATGGTTGCTTGGT	20	60,35	45	80
ADGRF5	ENST00000265417.7	hADGRF5_Fw1	TGAACAGCCTCAGTTTTCCA	20	59,41	45	hADGRF5_Rv1	GCAGGAGCACCAGATTTCAT	20	60,23	50	116
COMTD1	ENST00000372538.7	hCOMTD1_Fw1	AACTGCTCCGCCTACTACGA	20	60,04	55	hCOMTD1_Rv1	GTCCCCTTTCGGAGGTTG	18	60,44	61,11	111
RGPD4	ENST00000408999.3	hRGPD4_Fw1	GGTGGAAAGAGCAGCAAAAC	20	59,86	50	hRGPD4_Rv1	TCCATCCATCTTCACCTTCA	20	59,01	45	92
CLDN5	ENST00000403084.1	hCLDN5_Fw1	CTGGACCACAACATCGTGAC	20	60	55	hCLDN5_Rv1	CACCGAGTCGTACACTTTGC	20	59,36	55	105
DACH1	ENST00000611519.4	hDACH1_Fw1	AACCGCTGCAAACTCATCTC	20	60,41	50	hDACH1_Rv1	CTTAGGAGGCCTTCCAGGTC	20	60,2	60	90
ZFPM1	ENST00000319555.7	hZFPM1_Fw1	CTGGAAGGACAGGAACCAGA	20	60,23	55	hZFPM1_Rv1	CTCTGCCCATCCTGCAC	17	58,77	64,71	101
BACT	ENST00000331789,9	hACTBEx2FW1	GTCTTCCCCTCCATCGTG	18	58.2	61,1	hACTBEx3REV1	TCTTGCTCTGGGCCTCGT	18	58,2	61,1	97
HMBS	ENST00000278715.7	hHMBS_Fw2	CACCCACACACAGCCTACTTT	21	60,08	52,38	hHMBS_Rv2	GTACCCACGCGAATCACTCT	20	60,14	55	107

Supplementary Table 4. Top 15 hits *in-silico* off-target prediction miHTT-451 guide strand by siSPOTR

Number	Gene ID	Gene	TPOTS	8mer	7mer-M8	7mer-1A
**1**	80830	*APOL6*	0.596	2	3	1
**2**	79628	*SH3TC2*	0.508	1	4	1
**3**	2849	*GPR26*	0.434	1	2	2
**4**	51208	*CLDN18*	0.391	2	1	0
**5**	8828	*NRP2*	0.386	2	0	1
**6**	5241	*PGR*	0.385	2	0	1
**7**	340533	*KIAA2022*	0.367	1	2	1
**8**	775	*CACNA1C*	0.367	1	2	1
**9**	3977	*LIFR*	0.366	1	2	1
**10**	80034	*CSRNP3*	0.362	1	1	2
**11**	79068	*FTO*	0.355	1	0	3
**12**	257194	*NEGR1*	0.322	2	0	0
**13**	10492	*SYNCRIP*	0.321	2	0	0
**14**	6489	*ST8SIA1*	0.321	2	0	0
**15**	23007	*PLCH1*	0.321	2	0	0

Supplementary methods

Probability of Off-Target Score (POTS)

Provides summary information on the total number of predicted off-targets and the distribution of the various "site-types" found in those predicted targets. The POTS value is calculated by analyzing the potential off-target genes for seed matches conforming to known endogenous microRNA targeting rules. "8mer" sites tend to be the most potent followed by (in decreasing order) 7mer-M8, 7mer-1A and 6mer (https://sispotr.icts.uiowa.edu/sispotr/tools.html).

Potential off-target list

A list of probable off-target genes rank-ordered by individual transcript Probability of Off-Target Score (tPOTS). tPOTS was calculated based on the number and type of seed matches found in that transcript. "8mer" sites tend to be the most potent followed by (in decreasing order) 7mer-M8, 7mer-1A and 6mer (https://sispotr.icts.uiowa.edu/sispotr/tools.html).
